# Supplementary material for: Primary tumors release ITGBL1-rich extracellular vesicles to promote distal metastatic tumor growth through fibroblast-niche formation
Source: Nat Commun. 2020 Mar 5;11:1211. doi: 10.1038/s41467-020-14869-x (PMC7058049; doi:10.1038/s41467-020-14869-x)
Supplement: Supplementary file 1 — Supplementary Information [file 41467_2020_14869_MOESM1_ESM.pdf]

Supplementary Information

Primary tumors release ITGBL1-rich extracellular vesicles to promote distal metastatic tumor growth through fibroblast-niche formation

Ji et al.

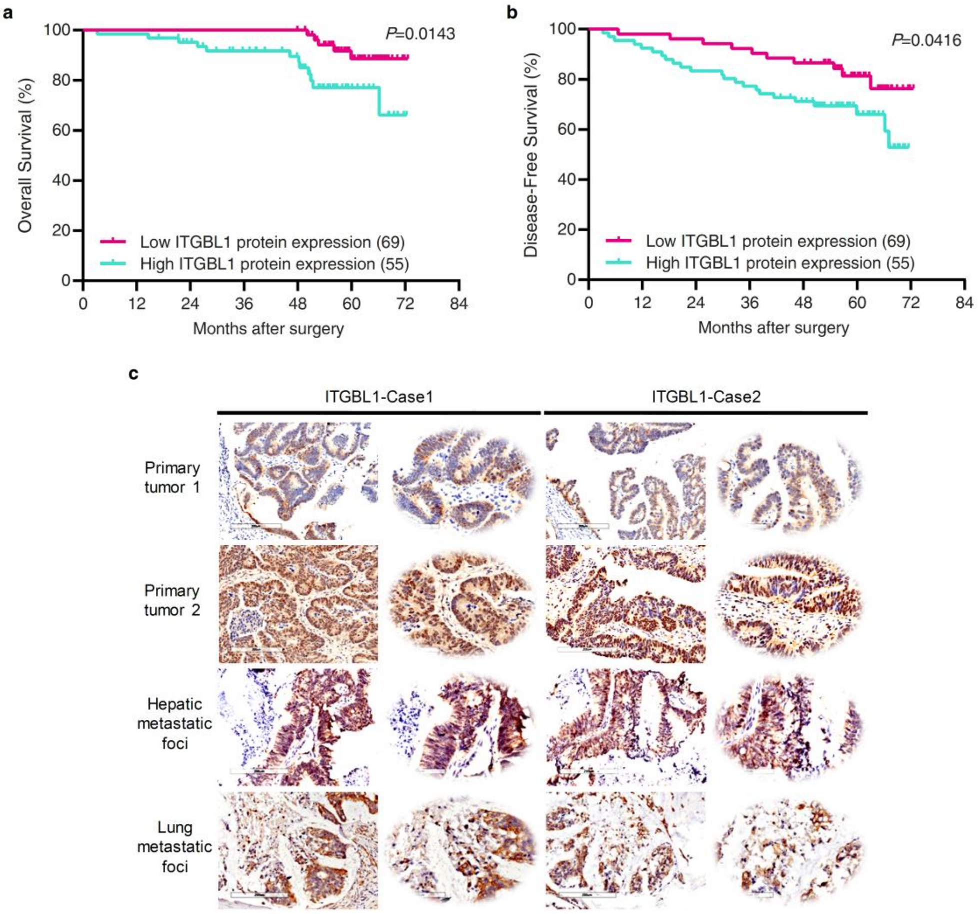

Supplementary Figure 1. Correlation of ITGBL1 protein levels with CRC prognosis. a, **b** Kaplan-Meier analyses of the correlations between ITGBL1 protein expression levels and OS or DFS in 124 CRC patients, and the median expression levels were used as the cutoff. Survival data were analyzed by the Kaplan-Meier method and log-rank test. **c** Immunohistochemical analysis of ITGBL1 protein in representative CRC and metastatic lung and liver tissues. Representative case 1 and case 2 were analyzed. Primary tumor I (without paired metastatic tissues) had low expression of ITGBL1, and primary tumor II (with paired hepatic and lung metastatic tissues) had high expression of ITGBL1 (scale bars, 200 and 50 mm, respectively). Each experiment was performed at least in triplicate.

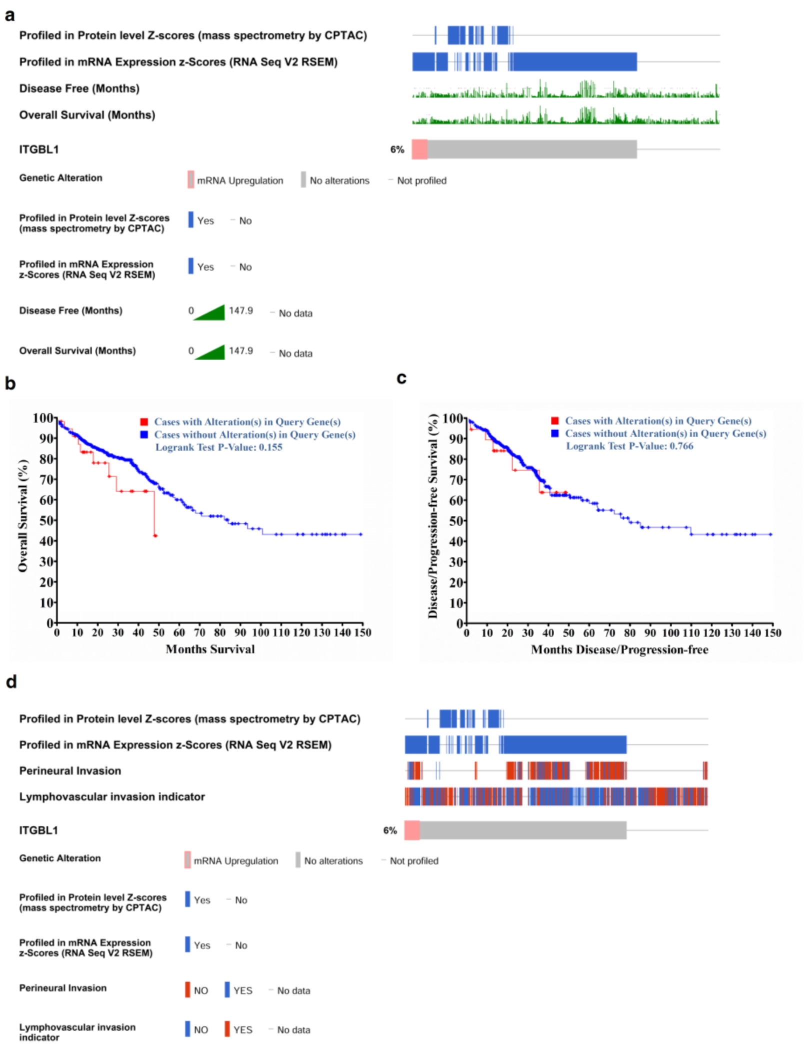

Supplementary Figure 2. Database mining of ITGBL1 expression in TCGA dataset. a-c mRNA expression of *ITGBL1* in 467 CRC primary tissues from TCGA (The Cancer Genome Atlas) dataset, and its association with CRC prognosis, including overall survival (OS) and disease-free survival (DFS). **d** Protein expression of ITGBL1 in 90 CRC primary tissues from TCGA dataset, which was characterized by mass spectrometry, and its association with lymphovascular invasion of CRC.

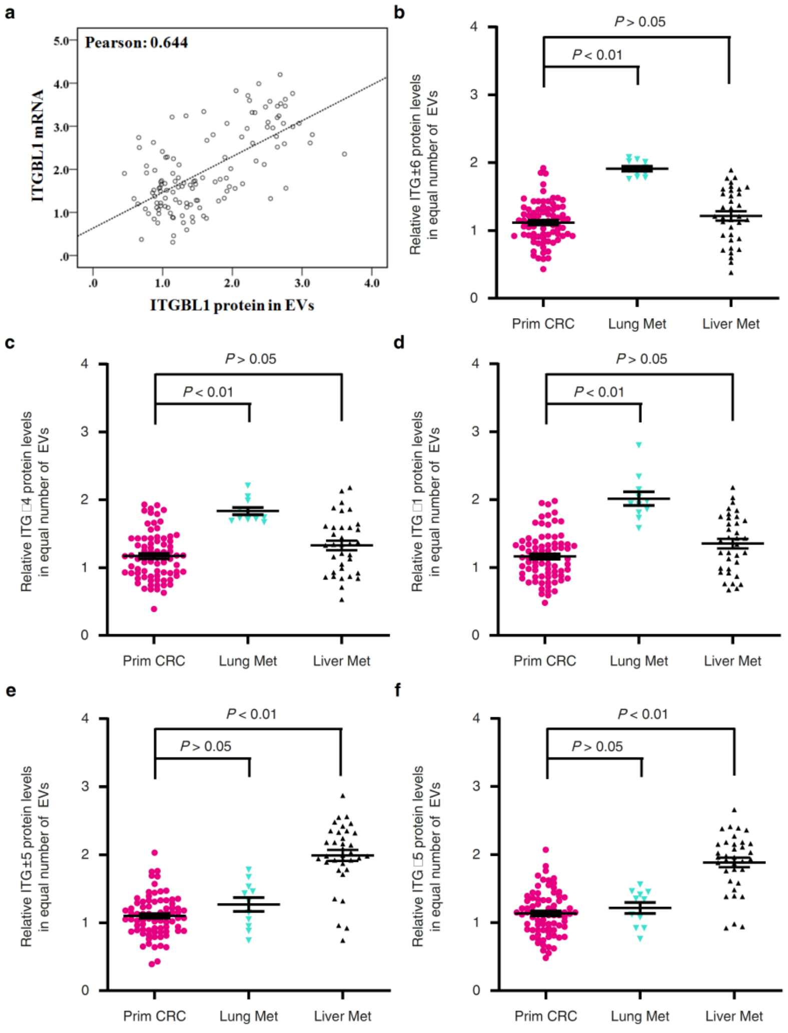

Supplementary Figure 3. Relative ITGBL1 levels in the plasma EVs of CRC patients and healthy volunteers. a Pearson correlations analysis between *ITGBL1* mRNA expression in 124 CRC tissues and ITGBL1 EVs expression in the plasma of 124 CRC patients. Pearson correlation coefficient was used to evaluate the correlation. Pearson correlation coefficient: 0.8-1.0, most strong correlation; 0.6-0.8, strong correlation; 0.4-0.6, middle correlation; 0.2-0.4, weak correlation; 0.0-0.2, no correlation. **b-f** Relative ITGα6, ITGβ4, ITGβ1, ITGα5 and ITGβ5 levels in the plasma EVs of post-surgical, primary and metastatic sites, comparing to primary sites of non-metastatic CRC patients. Primary CRC (78 cases): plasma from CRC patients without paired metastatic tissues, Lung Met (11 cases): plasma from CRC patients with paired lung metastatic tissues, Liver Met (35 cases): plasma from CRC patients with paired liver metastatic tissues. Each experiment was performed at least in triplicate and the

results are shown as mean ±SD. Student's t-test was used to analyze the data. *$p < 0.05$; **$p < 0.01$.

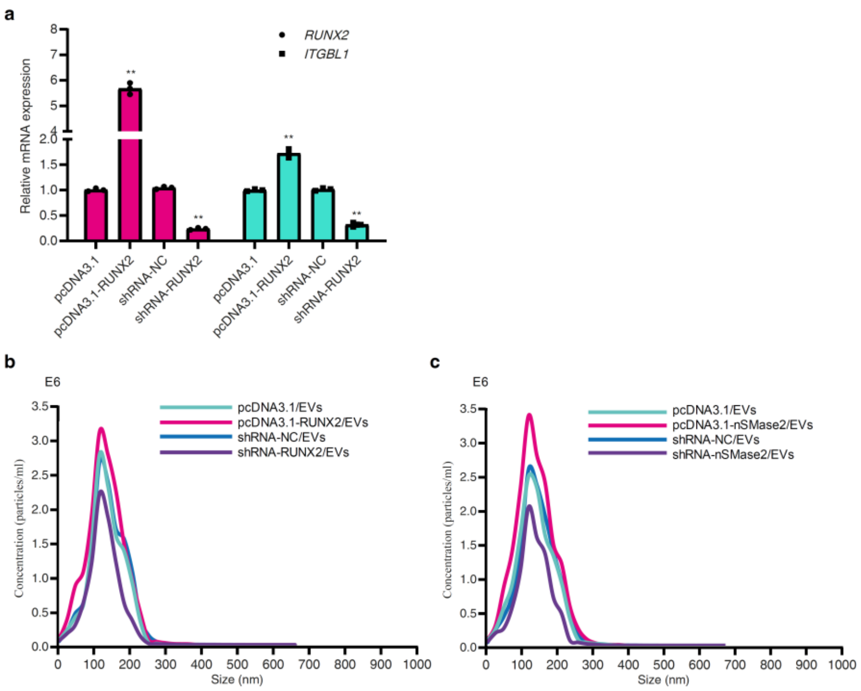

Supplementary Figure 4. The effect of RUNX2 and nSMase2 on the transcription and EVs secretion of ITGBL1. a RUNX2 was silent or overexpressed to observe its effect on the mRNA expression of ITGBL1. **b** RUNX2 was silent or overexpressed to observe its effect on the total numbers of EVs secreted by CRC SW620 cells using LM10 nanoparticle characterization system. Technical triplicates were analyzed. **c** nSMase2 was silent or overexpressed to observe its effect on the total numbers of EVs secreted by CRC SW620 cells using LM10 nanoparticle characterization system. Technical triplicates were analyzed. Each experiment was performed at least in triplicate and the results are shown as mean ±SD. Student's t-test was used to analyze the data. *$p < 0.05$; **$p < 0.01$.

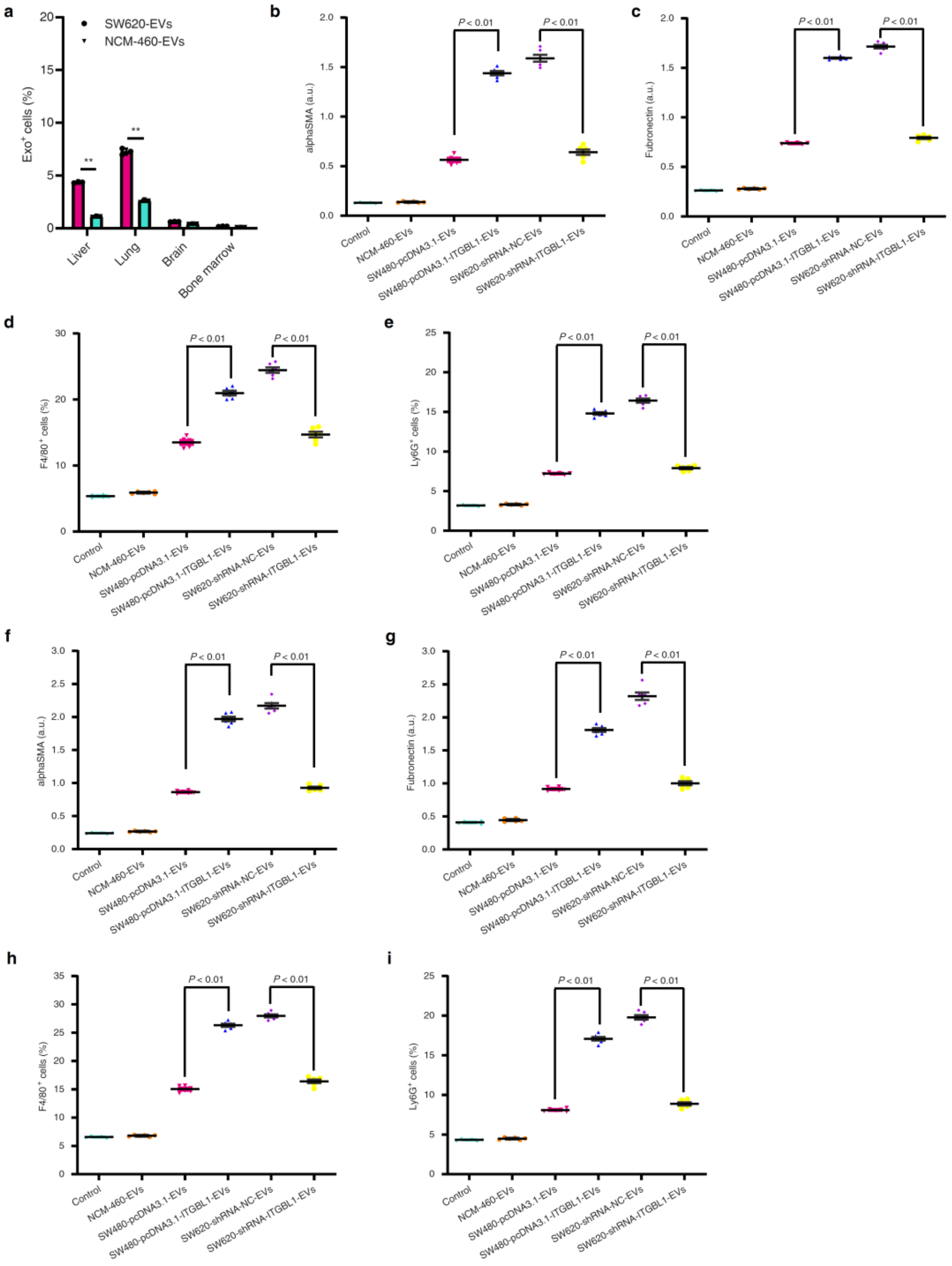

Supplementary Figure 5. EVs derived from ITGBL1-overexpressing CRC cells induced the liver and lung pre-metastatic niche formation. a Graph represents the quantification of EVs distribution in the liver, lung, brain, and bone marrow of mice educated with NCM-460-EVs and SW620-EVs by counting EVs-positive cells (for Fig 3**a**). An average of five random fields per sample were counted at 20 × magnification (three independent experiments, each with n = 3). **b-e** Immunofluorescence quantification analysis of α-SMA$^+$ hStCs, myofibroblasts, FN deposition, and recruitments of F4/80$^+$ macrophage and Ly6G$^+$

myeloid cells in the liver of mice educated with no EVs (control), NCM-460-EVs, SW480-pcDNA3.1-EVs, SW480-pcDNA3.1-ITGBL1-EVs, SW620-shRNA-NC-EVs, and SW620-shRNA-ITGBL1-EVs. **f-i** Immunofluorescence quantification analysis of α-SMA^{+} hStCs, myofibroblasts, FN deposition, and recruitments of F4/80^{+} macrophage and Ly6G^{+} myeloid cells in the lungs of mice educated with no EVs (control), NCM-460-EVs, SW480-pcDNA3.1-EVs, SW480-pcDNA3.1-ITGBL1-EVs, SW620-shRNA-NC-EVs, and SW620-shRNA-ITGBL1-EVs. Each experiment was performed at least in triplicate. The data was presented as the mean values ± SEM. Student's t-test was used to analyze the data. *$p < 0.05$; **$p < 0.01$.

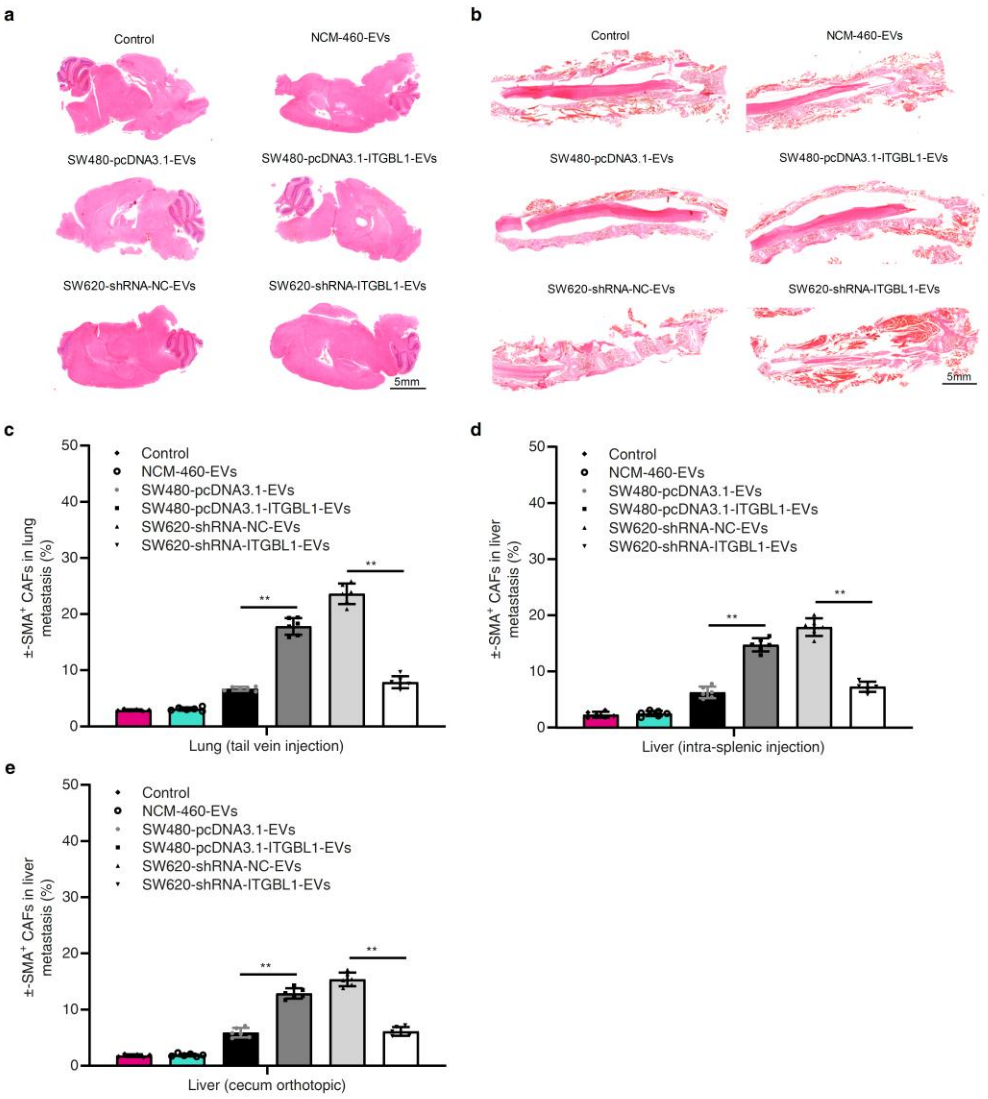

Supplementary Figure 6. Hematoxylin and eosin (H&E) staining of brain and bone tissue sections from indicated mice. a Representative pictures of H&E staining of brain tissue sections from indicated mice in Fig 4**e**. Scale bar, 5 mm. **b** Representative pictures of H&E staining of bone tissue sections from indicated mice in Fig 4**e**. Scale bar, 5 mm. **c-e** Quantitative analysis of fibroblast markers (α-SMA) in the CAFs isolated from the lung and liver metastatic tissues of indicated mice in Fig 4**a**, Fig 4**c**, and Fig 4**e**. Each experiment was performed at least in triplicate and all the data are shown as mean ± SD. Student's t-test was used to analyze the data. *p < 0.05; **p < 0.01.

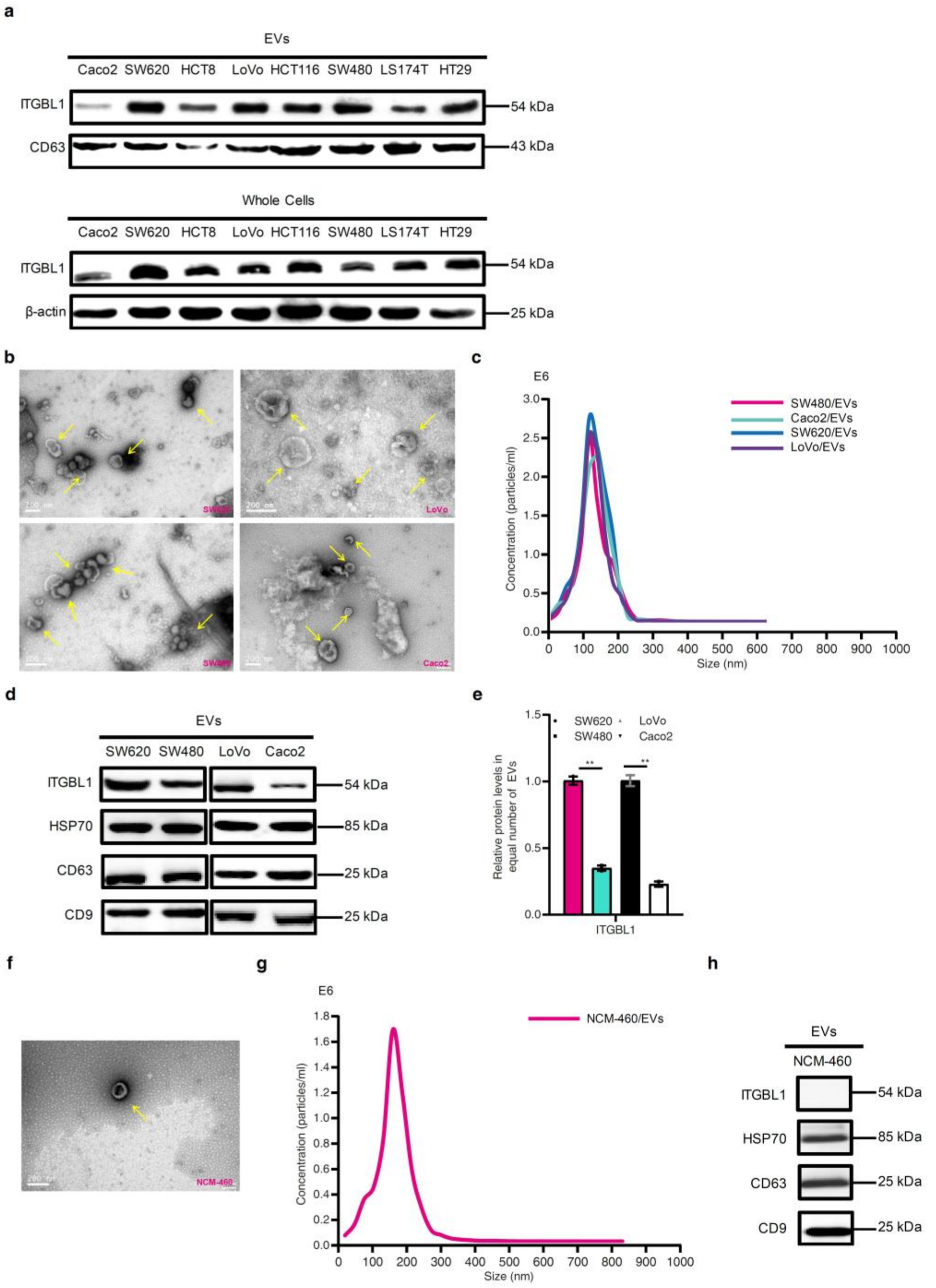

Supplementary Figure 7. Basal protein expressions of ITGBL1 in the intracellular and EVs of CRC cell lines. a Protein expressions of ITGBL1 in the intracellular and EVs of CRC cell lines. **b** CRC cells-derived EVs from SW620, SW480, LoVo and Caco2 cells were analyzed for phenotype (purity and shape) by electron microscopy, and the yellow arrows indicate the representative EVs. Scale bar, 200 nm. **c** CRC cells-derived EVs were analyzed for size and particle number by LM10 nanoparticle characterization system (NanoSight, Malvern Instruments). **d, e** Immunoblotting and quantitative assays of ITGBL1, HSP70,

CD63 and CD9 in different CRC cells, including SW620, SW480, LoVo and Caco2 cells. **f** EVs from normal colonic epithelial cells NCM-460 were analyzed for phenotype (purity and shape) by electron microscopy, and the yellow arrows indicate the representative EVs. Scale bar, 200 nm. **g** NCM-460 EVs were analyzed for size and particle number by LM10 nanoparticle characterization system. **h** Immunoblotting assays of ITGBL1, HSP70, CD63 and CD9 in NCM-460 cells. Each experiment was performed in triplicate. All the data are shown as mean ± SD. Student's t-test was used to analyze the data. *$p < 0.05$; **$p < 0.01$.

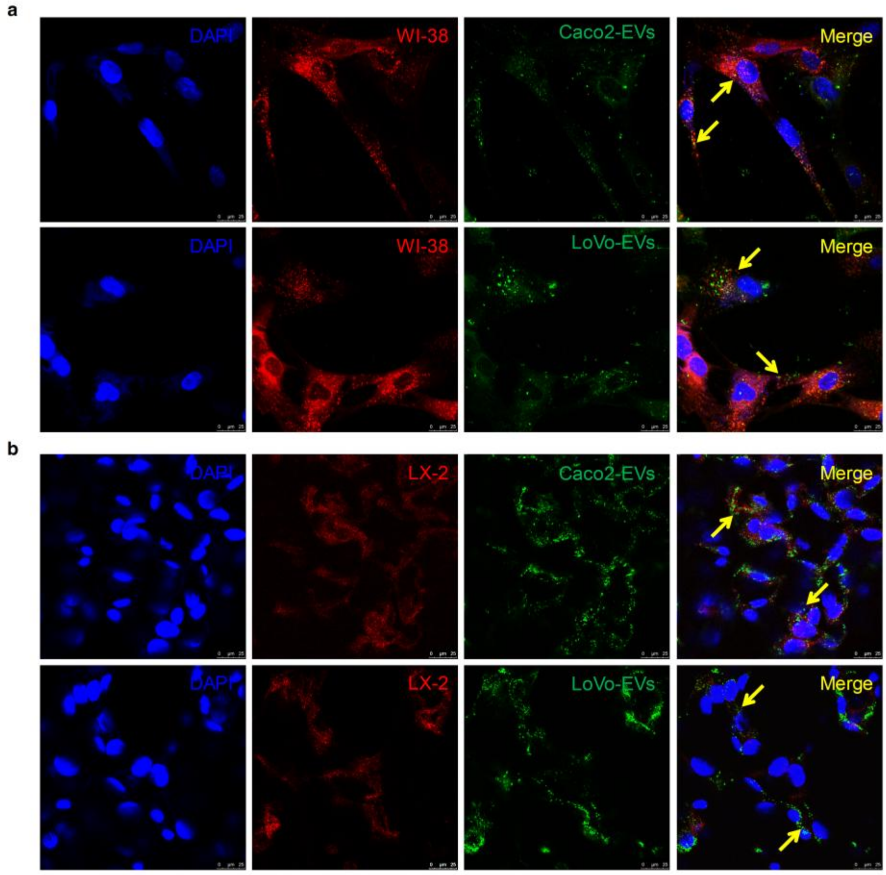

Supplementary Figure 8. Tracing PKH26-labeled CRC cells-derived EVs in lung fibroblasts WI-38 and hepatic stellate cells LX-2. a Confocal imaging showed the delivery of PKH67-labeled EVs (green) to PKH26-labeled WI-38 (red). EVs were derived from Caco2 or LoVo cells. Yellow arrows represented delivered EVs in the representative images. Scale bar, 25 μm. **b** Confocal imaging showed the delivery of PKH67-labeled EVs (green) to PKH26-labeled LX-2 (red). EVs were derived from Caco2 or LoVo cells. Yellow arrows represented delivered EVs in the representative images. Scale bar, 25 μm. Each experiment was performed in triplicate.

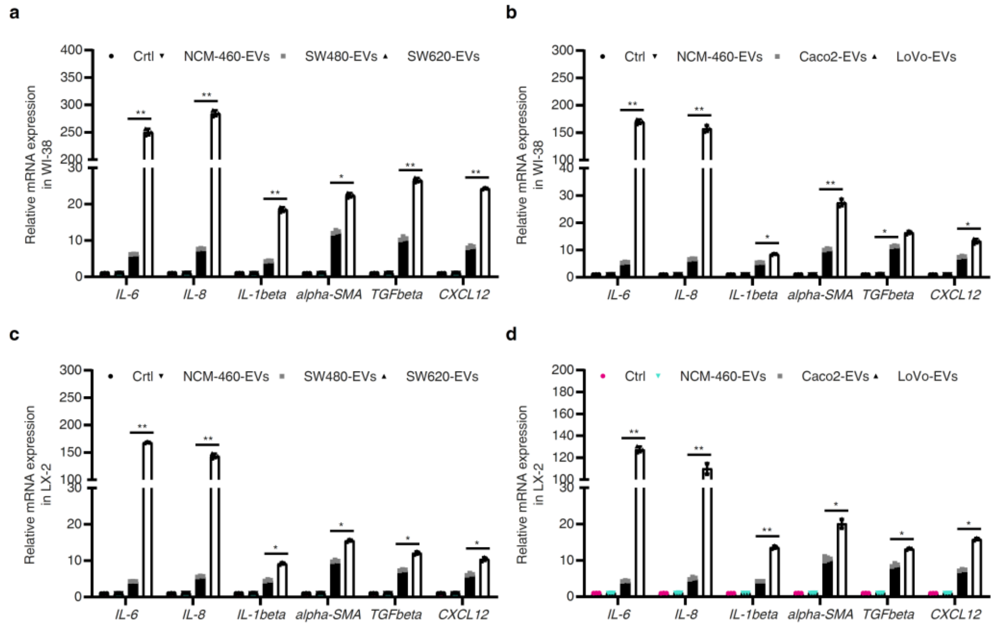

Supplementary Figure 9. EVs secreted from high-metastatic CRC cells promoted the expression of cytokine. a-d qRT-PCR analysis of indicated gene (*IL-6*, *IL-8*, *IL-1β*, *α-SMA*, *TGF-β*, *CXCL12*) expressions in WI-38 or LX-2 treated with EVs secreted from normal colonic epithelial cells NCM-460 and different CRC cells (SW480, SW620, Caco2, LoVo), and the control was the WI-38 or LX-2 cells without EVs treatment. Each experiment was performed at least in triplicate and the results are shown as mean ±SD. Student's t-test was used to analyze the data. *$p < 0.05$; **$p < 0.01$.

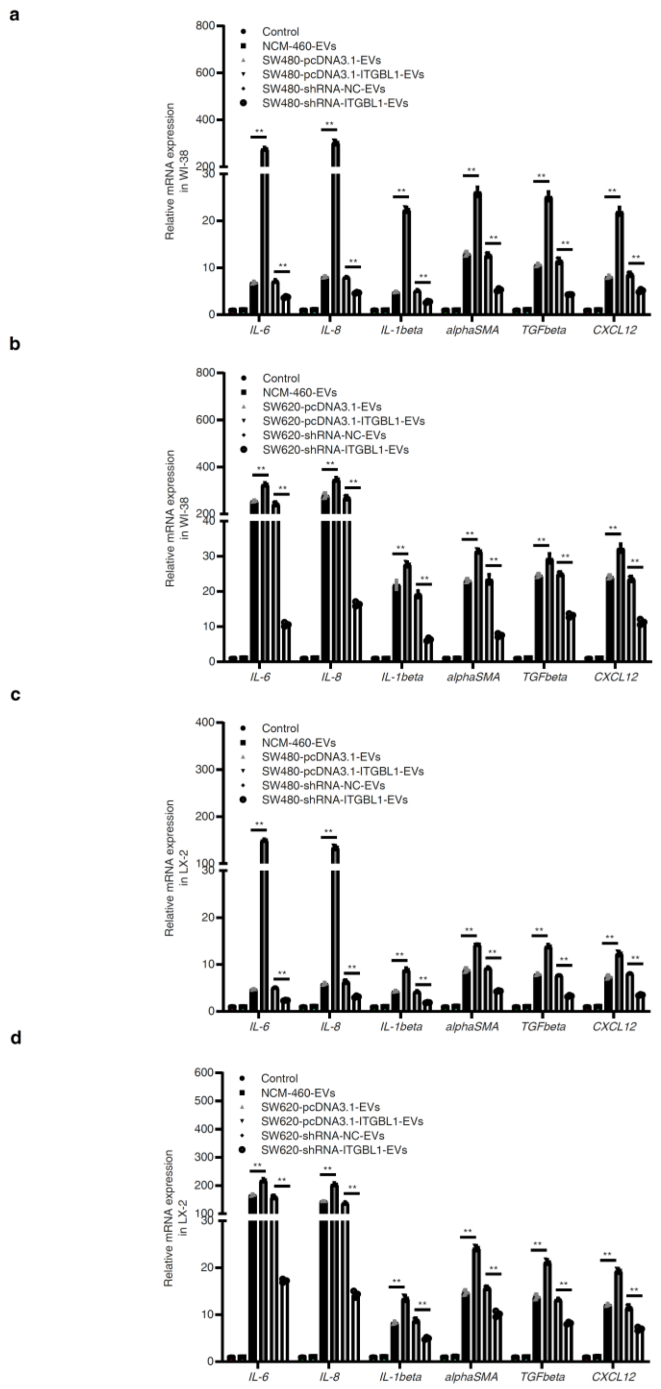

Supplementary Figure 10. High ITGBL1-loaded EVs secreted from high-metastatic CRC cells promoted the expression of cytokine. a qRT-PCR analysis of indicated gene (*IL-6, IL-8, IL-1β*, *α-SMA*, *TGF-β*, *CXCL12*) expressions in WI-38 cells treated with EVs derived from NCM-460 cells and ITGBL1-overexpressing or -silent CRC SW480 cells relative to their respective controls. The blank control was the WI-38 cells without EVs treatment. **b** qRT-PCR analysis of indicated gene (*IL-6, IL-8, IL-1β*, *α-SMA*, *TGF-β*, *CXCL12*) expressions in WI-38 cells treated with EVs derived from NCM-460 cells and

ITGBL1-overexpressing or -silent CRC SW620 cells relative to their respective controls. The blank control was the WI-38 cells without EVs treatment. **c** qRT-PCR analysis of indicated gene (*IL-6*, *IL-8*, *IL-1β*, *α-SMA*, *TGF-β*, *CXCL12*) expressions in LX-2 cells treated with EVs derived from NCM-460 cells and ITGBL1-overexpressing or -silent CRC SW480 cells relative to their respective controls. The blank control was the LX-2 cells without EVs treatment. **d** qRT-PCR analysis of indicated gene (*IL-6*, *IL-8*, *IL-1β*, *α-SMA*, *TGF-β*, *CXCL12*) expressions in LX-2 cells treated with EVs derived from NCM-460 cells and ITGBL1-overexpressing or -silent CRC SW620 cells relative to their respective controls. The blank control was the LX-2 cells without EVs treatment. Each experiment was performed at least in triplicate and the results are shown as mean ±SD. Student's t-test was used to analyze the data. *$p < 0.05$; **$p < 0.01$.

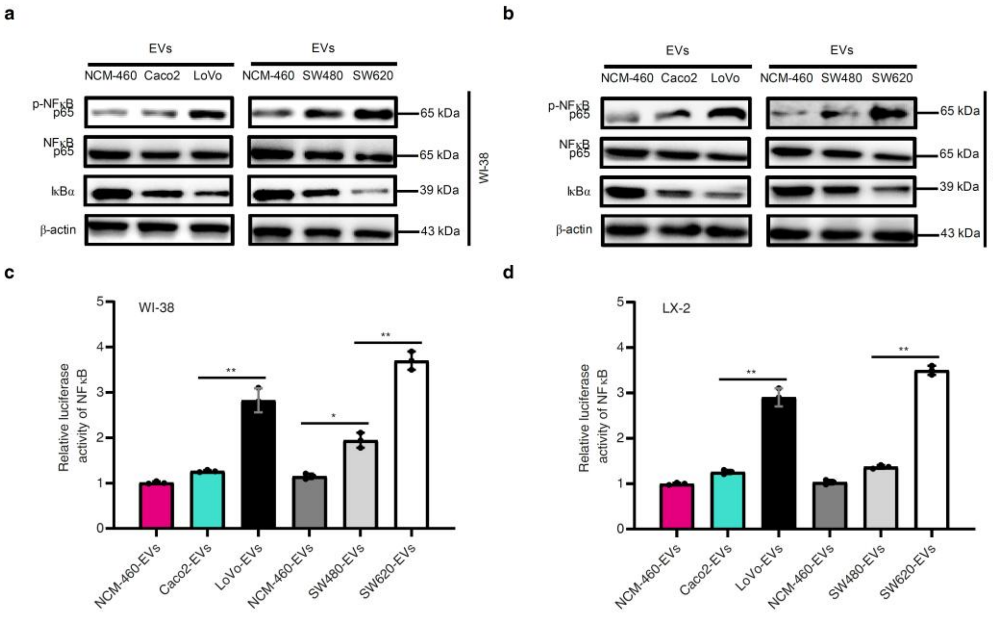

Supplementary Figure 11. EVs from high-metastatic CRC cells activated NF-κB signaling pathway. a, b Immunoblotting assays of indicated proteins (p-NF-κB, NF-κB, IκBα, β-actin) in WI-38 or LX-2 treated with EVs from different CRC cells and normal colonic epithelial cells NCM-460. **c, d** Relative luciferase activity of NF-κB in WI-38 or LX-2 treated with EVs from different CRC cells and normal colonic epithelial cells NCM-460. Each experiment was performed at least in triplicate and the results are shown as mean ±SD. Student's t-test was used to analyze the data. *$p < 0.05$; **$p < 0.01$.

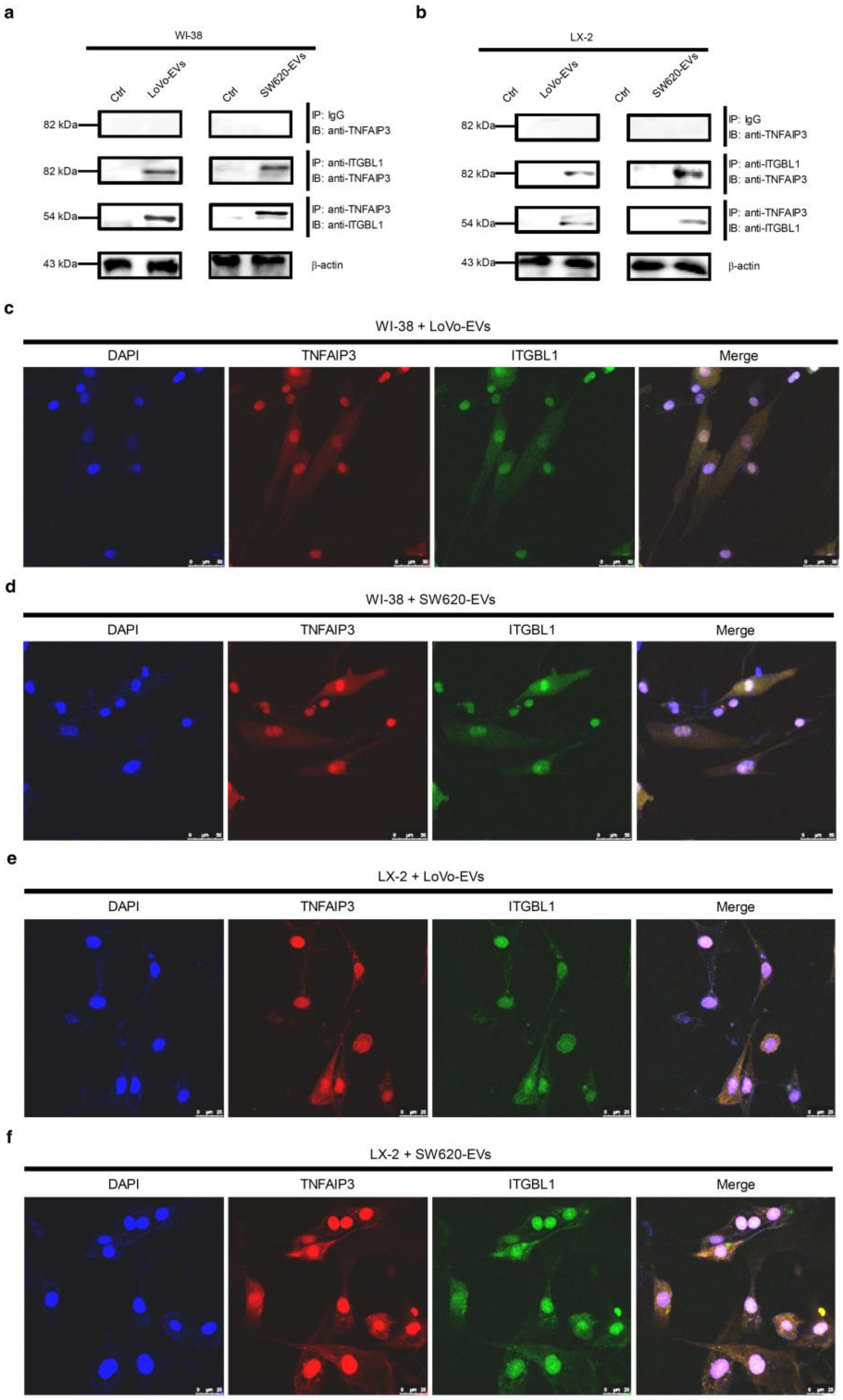

Supplementary Figure 12. Direct protein interaction between ITGBL1 and TNFAIP3 in the lung fibroblasts or hepatic stellate cells treated with ITGBL1-enriched EVs. a Co-IP in combination with western blot was performed to validate the interaction between ITGBL1 and TNFAIP3 in WI-38 cells treated with ITGBL1-enriched EVs from LoVo or SW620 cells. The blank control was the WI-38 cells without EVs treatment. **b** Co-IP in combination with

western blot was performed to validate the interaction between ITGBL1 and TNFAIP3 in LX-2 cells treated with ITGBL1-enriched EVs from LoVo or SW620 cells. The blank control was the LX-2 cells without EVs treatment. **c-d** Immunofluorescence detection of the co-location of ITGBL1 and TNFAIP3 in WI-38 cells treated with ITGBL1-enriched EVs from LoVo or SW620 cells. Scale bar, 50 μm. **e-f** Immunofluorescence detection of the co-location of ITGBL1 and TNFAIP3 in LX-2 cells treated with ITGBL1-enriched EVs from LoVo or SW620 cells. Each experiment was performed at least in triplicate and the results are shown as mean ±SD. Student's t-test was used to analyze the data. *p < 0.05; **p < 0.01.

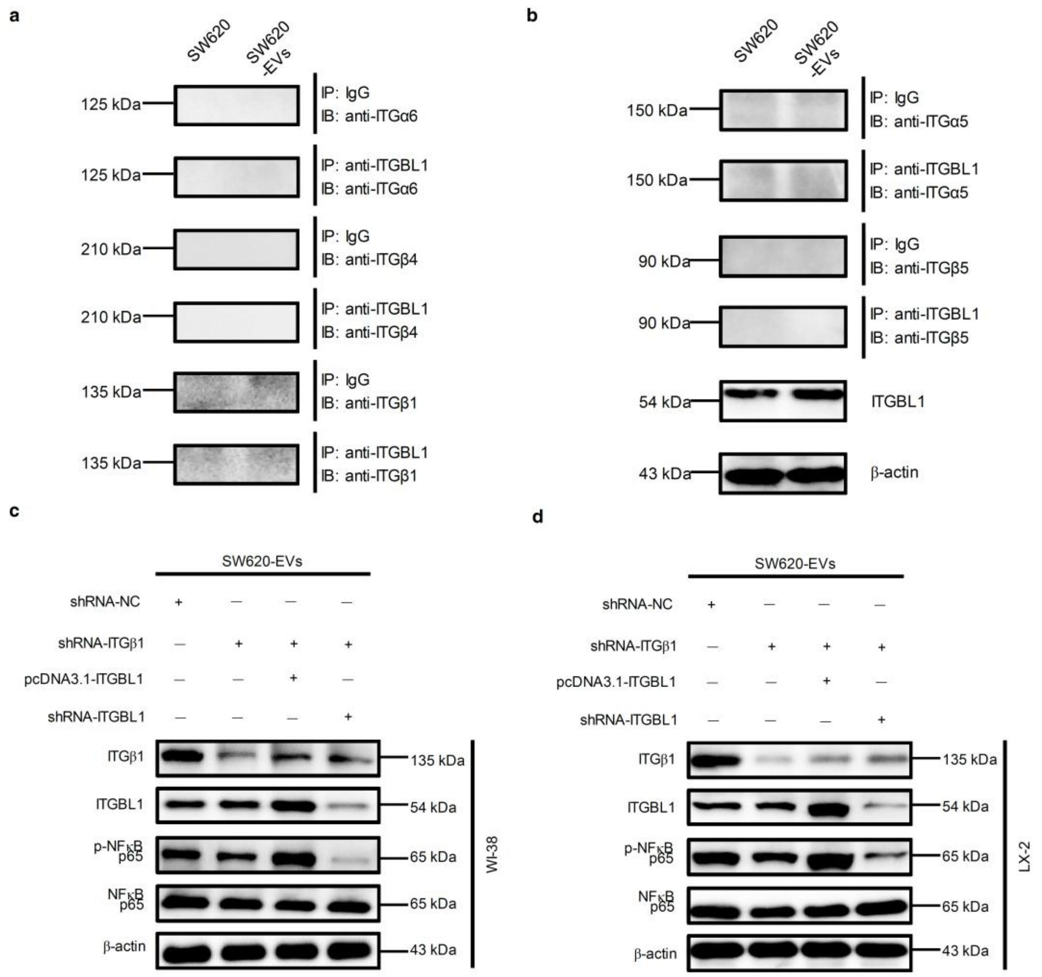

Supplementary Figure 13. ITGBL1 did not bind to other integrins, and ITGβ1 barely affect the regulatory effect of ITGBL1 on NF-κB signaling pathway. a, b Co-IP in combination with western blot was performed to observe the interaction between ITGBL1 and integrins of different subtype (ITGα6, ITGβ4, ITGβ1, ITGα5 and ITGβ5) in SW620 cells or SW620-derived EVs. **c, d** Immunoblotting assays of indicated proteins (ITGβ1, ITGBL1, p-NF-κB, NF-κB, β-actin) in WI-38 or LX-2 treated with EVs from ITGβ1-silent or/and ITGBL1-overexpressing or ITGBL1-silent SW620 cells. EVs from shRNA-NC SW620 cells were used as the controls. Each experiment was performed at least in triplicate.

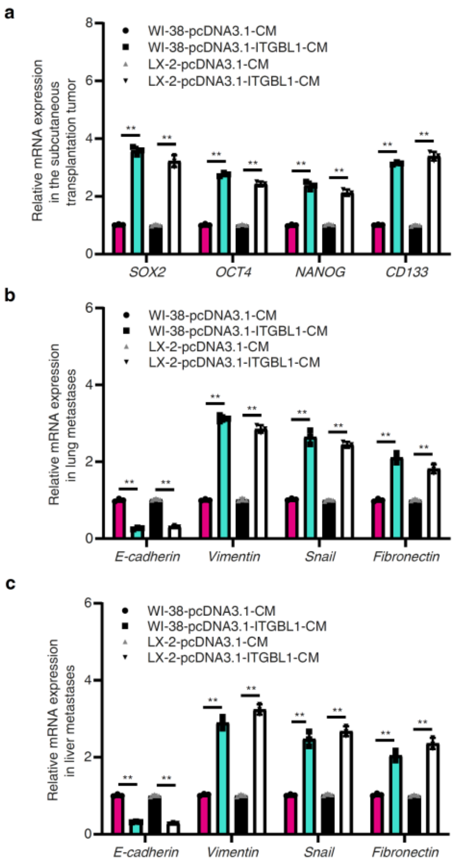

Supplementary Figure 14. qRT-PCR analysis of stemness-associated genes expression and EMT-associated genes expression in tumor tissues from indicated mice. a qRT-PCR analysis of stemness-associated genes expression in the subcutaneous transplantation tumors from indicated mice in Fig 6**i**. **b** qRT-PCR analysis of EMT-associated genes expression in the lung metastases from indicated mice in Fig 7**e**. **c** qRT-PCR analysis of EMT-associated gene expression in the liver metastases from indicated mice in Fig 7**f**. Each experiment was performed at least in triplicate and the results are shown as mean ±SD. Student’s t-test was used to analyze the data. *p < 0.05; **p < 0.01.

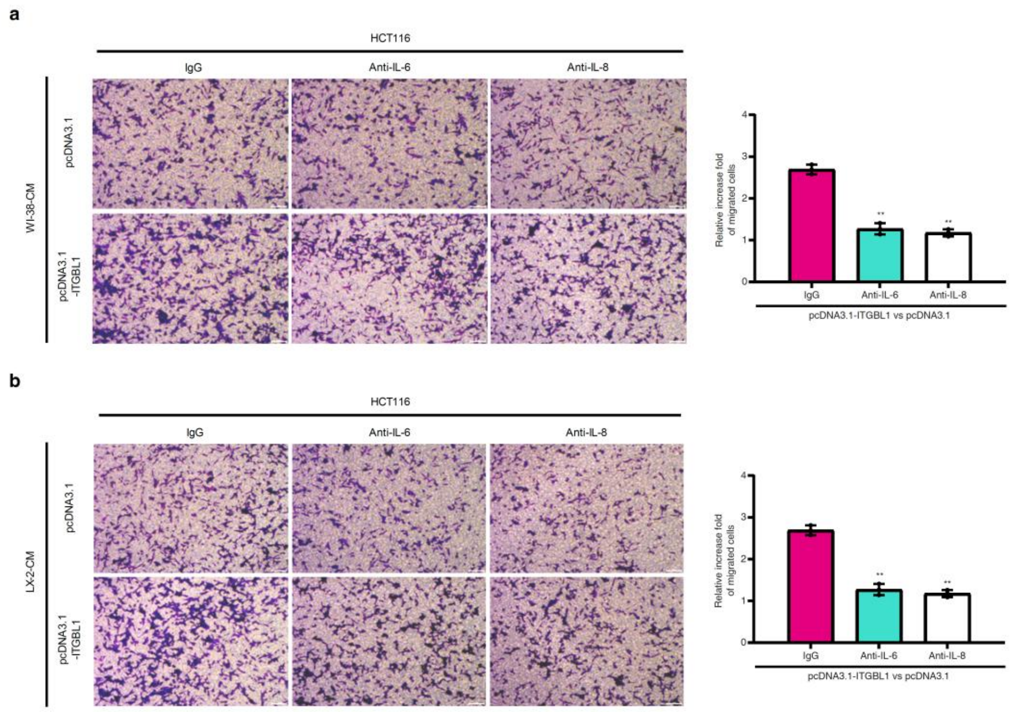

Supplementary Figure 15. Relative migration ability of HCT116 cells treated with indicated conditioned medium (CM) containing anti-IL-6/anti-IL-8 antibody or control IgG antibody. Representative images and quantitative results were shown in **a** and **b**. Scale bar, 150 μm. Each experiment was performed at least in triplicate and the results are shown as mean ±SD. Student's t-test was used to analyze the data. **p* < 0.05; ***p* < 0.01.

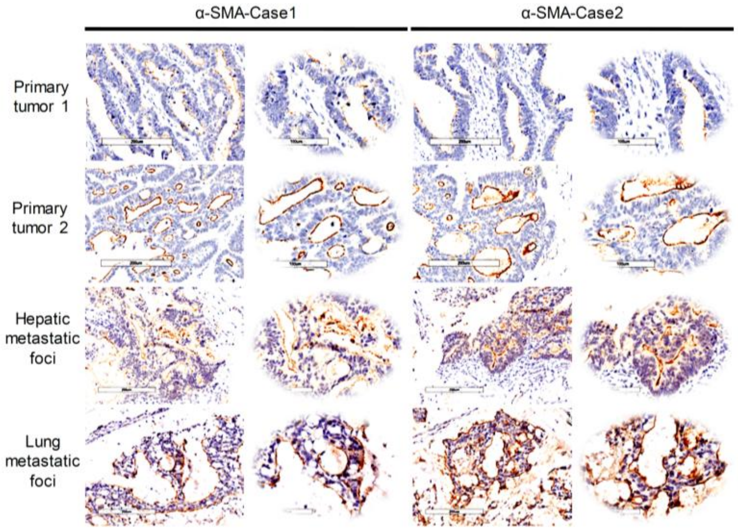

Supplementary Figure 16. Immunohistochemical analysis of fibroblast markers (α-SMA) in representative CRC and metastatic liver and lung tissues. Representative case 1 and case 2 were analyzed. Primary tumor I (without paired metastatic tissues) had low expression of α-SMA, and primary tumor II (with paired liver or lung metastatic tissues) had high expression of α-SMA (scale bars, 200 and 50 μm, respectively). Each experiment was performed at least in triplicate.

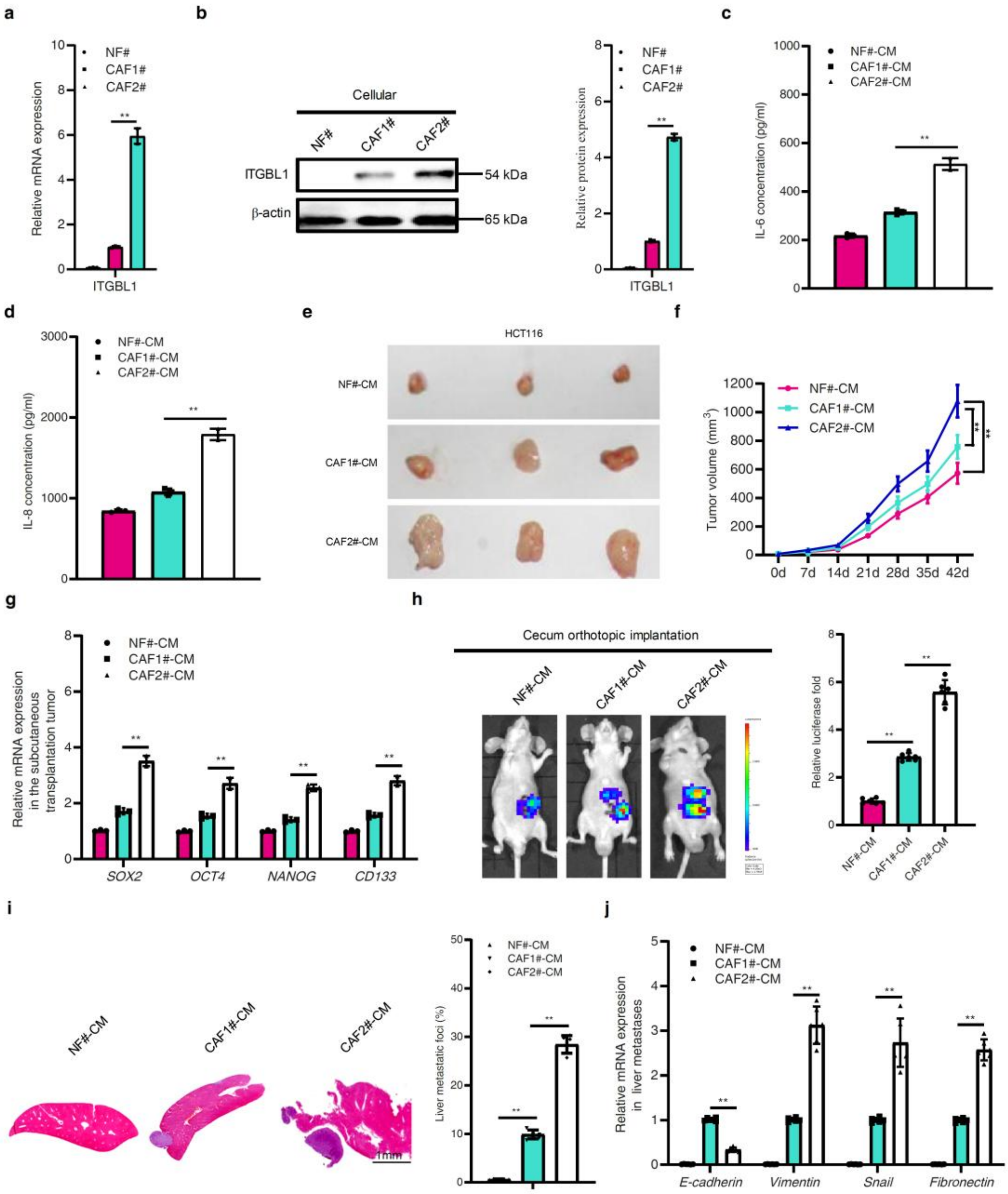

Supplementary Figure 17. Primary cancer-associated fibroblasts (CAFs) contribute to CRC progression *in vivo*. a qRT-PCR analysis of *ITGBL1* mRNA expression in the representative CAF1# (low *ITGBL1* mRNA) and CAF2# (high *ITGBL1* mRNA) from liver metastatic tissues of CRC patients or NFs (normal fibroblast) from normal liver. **b** Immunoblotting and quantitative assays of ITGBL1 proteins in CAF1#, CAF2# and NFs. β-Actin was used as the internal control. **c, d** ELISA assays on the IL-6 and IL-8 levels from the conditioned medium (CM) from primary cultured CAF1#, CAF2# and NFs. **e, f** Xenograft assays of HCT116 cells with indicated treatments were carried out on nude mice. HCT116 cells were pretreated with the conditioned medium (CM) from primary cultured CAF1#,

CAF2# and NFs. Representative tumors and tumors growth curves were shown. **g** qRT-PCR analysis of stemness-associated genes expression in the subcutaneous transplantation tumor from indicated mice in **e**. **h** Metastasis assays of HCT116 cells with indicated treatments performed on nude mice. HCT116 cells were pretreated with the conditioned medium of primary cultured CAF1#, CAF2# and NFs. Representative images and quantitative analysis of liver metastases of indicated mice were determined by luciferase-based bioluminescence imaging. For each group, 6 mice were used for quantification. **i** Representative pictures and quantitative results of H&E staining of liver tissue sections from indicated mice in **h**. Left panel: H&E images; right panel: the quantitative data for lung metastatic foci coverage. Scale bar, 1 mm. **j** qRT-PCR analysis of EMT-associated gene expression in the liver metastases from indicated mice in **h**. Each experiment was performed at least in triplicate and the results are shown as mean ±SD. Student's t-test was used to analyze the data. *$p < 0.05$; **$p < 0.01$.

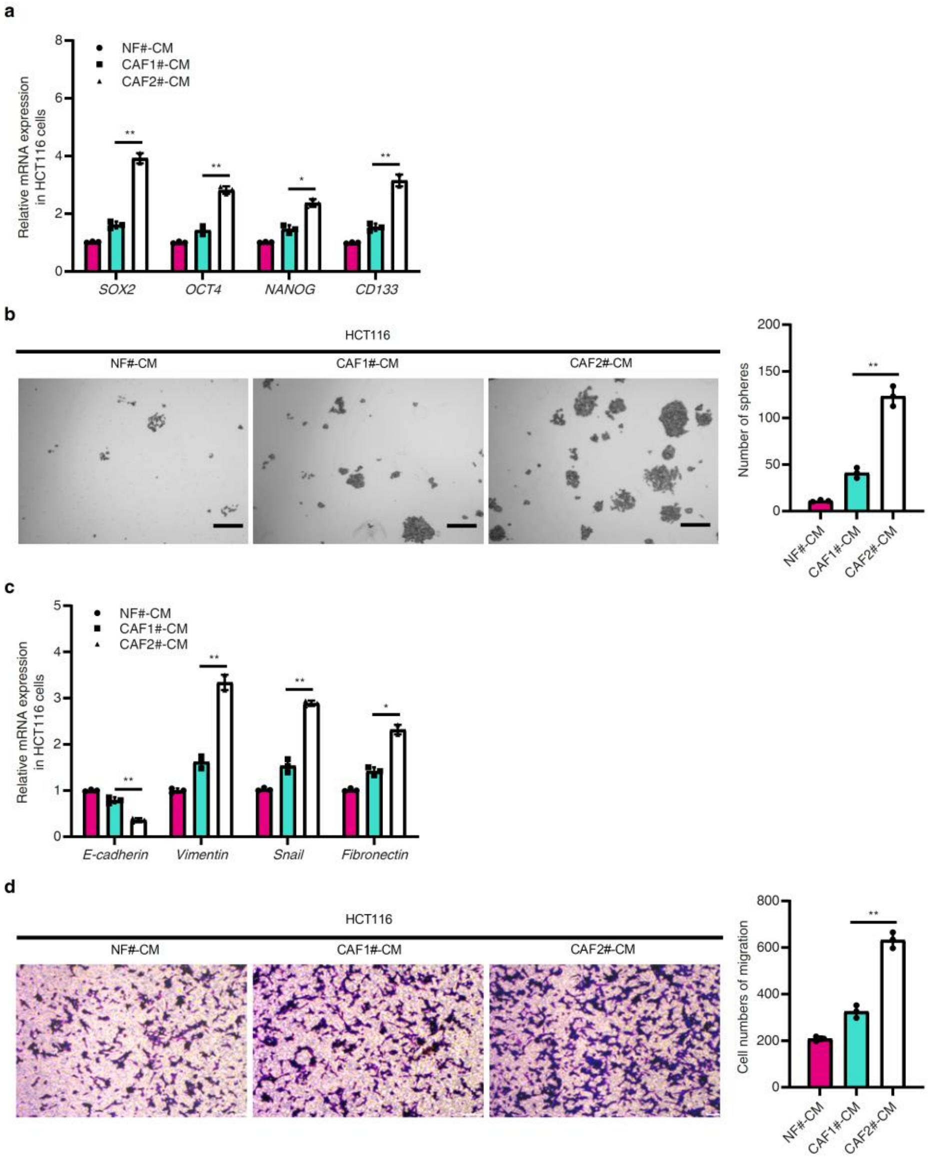

Supplementary Figure 18. Primary cancer-associated fibroblasts (CAFs) contribute to the spheroid formation and migration of CRC cells *in vitro*. a qRT-PCR analysis of stemness-associated genes expression in HCT116 cells pretreated with the conditioned medium (CM) from primary cultured CAF1#, CAF2# and NFs. **b** Spheroid formation and quantitative assay of HCT116 cells with indicated treatments. Representative images were represented and spheroid cells were counted. Scale bar, 150 μm. **c** qRT-PCR analysis of EMT-associated genes expression in HCT116 cells pretreated with the conditioned medium (CM) from primary cultured CAF1#, CAF2# and NFs. **d** Migration and quantitative assay of HCT116 cells with indicated treatments. Representative images were represented and migrated cells were counted. Scale bar, 150 μm. Each experiment was performed at least in

triplicate and the results are shown as mean ±SD. Student's t-test was used to analyze the data. *$p < 0.05$; **$p < 0.01$.

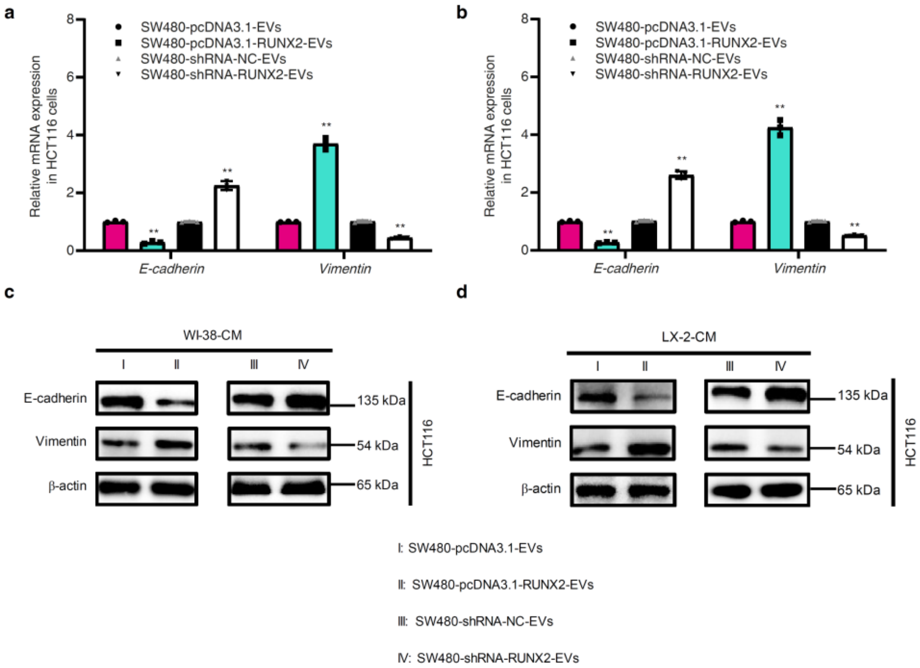

Supplementary Figure 19. High ITGBL1-loaded EVs secreted from high-metastatic CRC cells promoted the expression of cytokine. a qRT-PCR analysis of EMT-associated genes expression in HCT116 cells treated with WI-38-CM educated by EVs derived from RUNX2-overexpressing or -silent SW480 cells. **b** qRT-PCR analysis of EMT-associated genes expression in HCT116 cells treated with LX-2-CM educated by EVs derived from RUNX2-overexpressing or -silent SW480 cells. **c** Immunoblotting assays of EMT-associated protein expression in HCT116 cells treated with WI-38-CM educated by EVs derived from RUNX2-overexpressing or -silent SW480 cells. **d** Immunoblotting assays of EMT-associated protein expression in HCT116 cells treated with LX-2-CM educated by EVs derived from RUNX2-overexpressing or -silent SW480 cells. I: SW480-pcDNA3.1-EVs; II: SW480-pcDNA3.1-RUNX2-EVs; III: SW480-shRNA-NC-EVs; IV: SW480-shRNA-RUNX2-EVs. Each experiment was performed at least in triplicate and the results are shown as mean ±SD. Student's t-test was used to analyze the data. *$p < 0.05$; **$p < 0.01$.

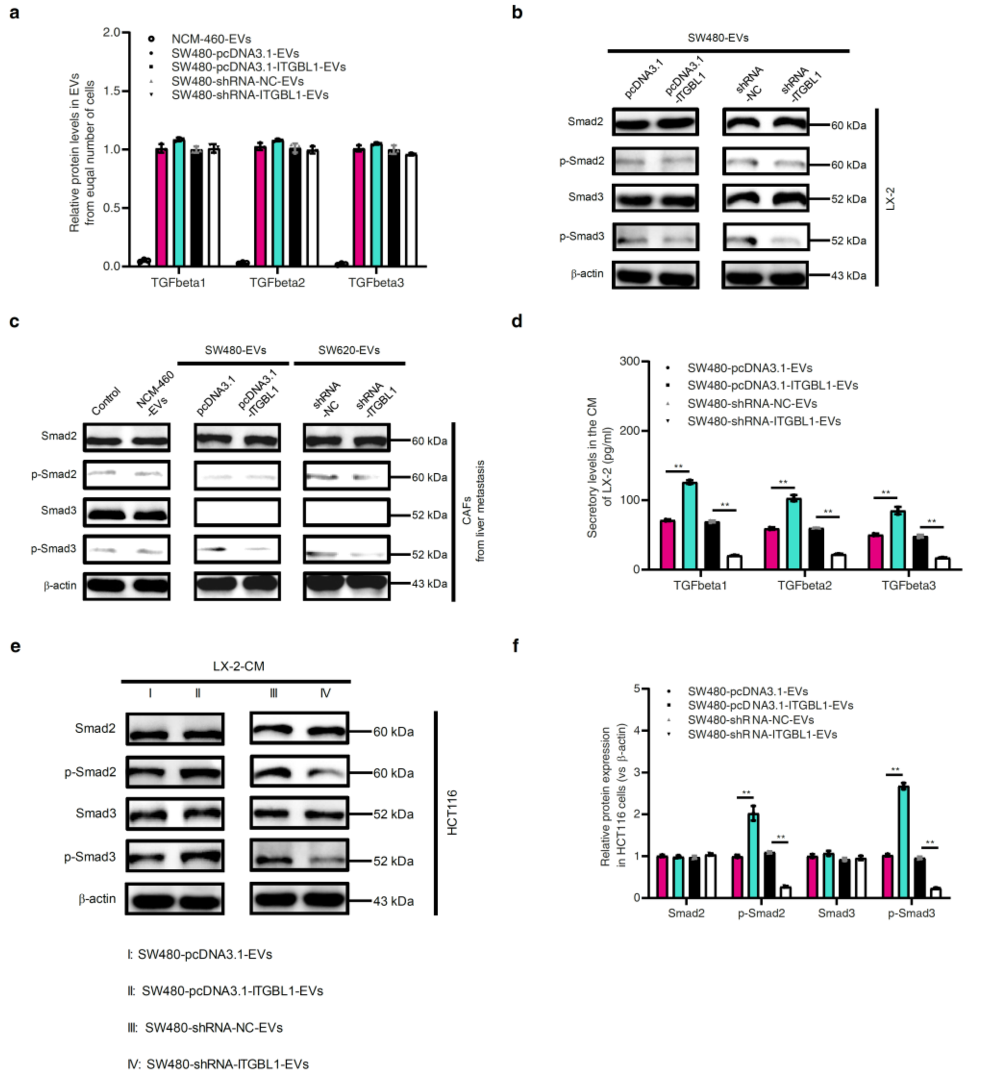

Supplementary Figure 20. TGF-β signaling is not influenced in the activated fibroblasts *in vitro* or CAFs *in vivo* upon indicated EVs treatment. a ELISA assays of TGFβ1, TGFβ2, and TGFβ3 protein levels in the EVs from NCM-460, SW480-pcDNA3.1, SW480-pcDNA3.1-ITGBL1, SW480-shRNA-NC and SW480-shRNA-ITGBL1 cells. **b** Immunoblotting assays of TGF-β/Smads signaling associated proteins (Smad2, p-Smad2, Smad3, p-Smad3) in LX-2 cells treated with EVs from indicated cells. **c** Immunoblotting assays of indicated proteins in the CAFs isolated from liver metastasis in Fig 4**e**. **d** ELISA assays of TGFβ1, TGFβ2, and TGFβ3 protein levels in the conditioned medium (CM) of LX-2 cells treated with EVs derived from ITGBL1-overexpressing or -silent SW480 cells, or respective controls. **e, f** Immunoblotting and quantitative assays of indicated proteins in HCT116 treated with conditioned medium (CM) from LX-2 educated with EVs derived from ITGBL1-overexpressing or -silent SW480 cells, or respective controls. I:

SW480-pcDNA3.1-EVs; II: SW480-pcDNA3.1-ITGBL1-EVs; III: SW480-shRNA-NC-EVs; IV: SW480-shRNA-ITGBL1-EVs. Each experiment was performed at least in triplicate and the results are shown as mean ±SD. Student's t-test was used to analyze the data. *$p < 0.05$; **$p < 0.01$.

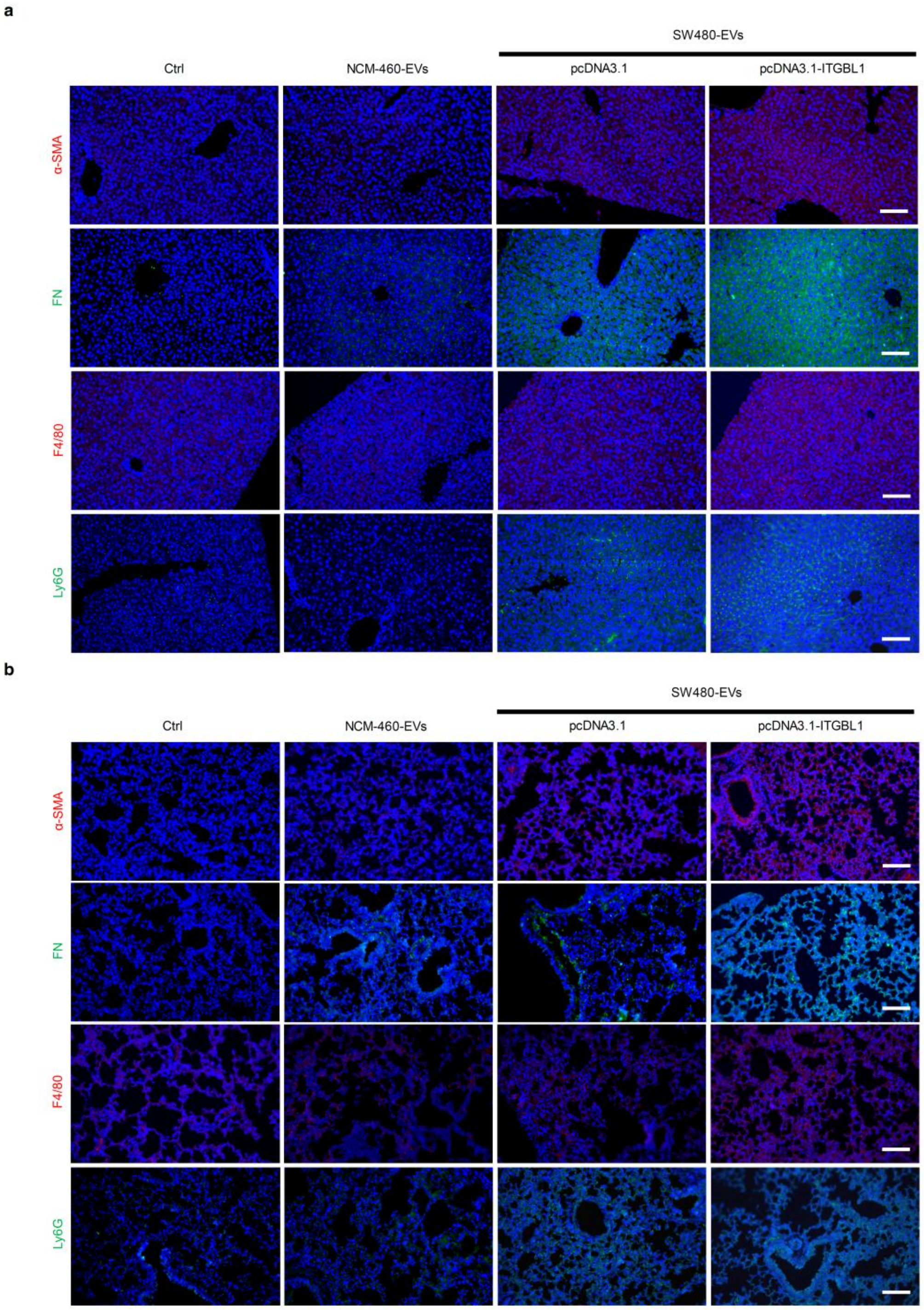

Supplementary Figure 21. EVs derived from ITGBL1-overexpressing CRC cells induce the liver and lung pre-metastatic niche formation in C57Bl/6 mice. a, b Representative immunofluorescence images of α-SMA and FN expression in arbitrary units (a.u.), F4/80$^+$ cells, and Ly6G$^+$ cells in the liver and lung of C57Bl/6 mice educated with no EVs (control), NCM-460-EVs, SW480-pcDNA3.1-EVs, and SW480-pcDNA3.1-ITGBL1-EVs. For α-SMA and F4/80 detection, secondary antibodies conjugated to Alexa Fluor 594 (Red) were used.

For FN and Ly6G detection, secondary antibodies conjugated to Alexa Fluor 488 (Green) were used. Nuclear staining was done with DAPI (40, 6-diamidino-2-phenylindole). Scale bar, 100 μm. Each experiment was performed at least in triplicate.

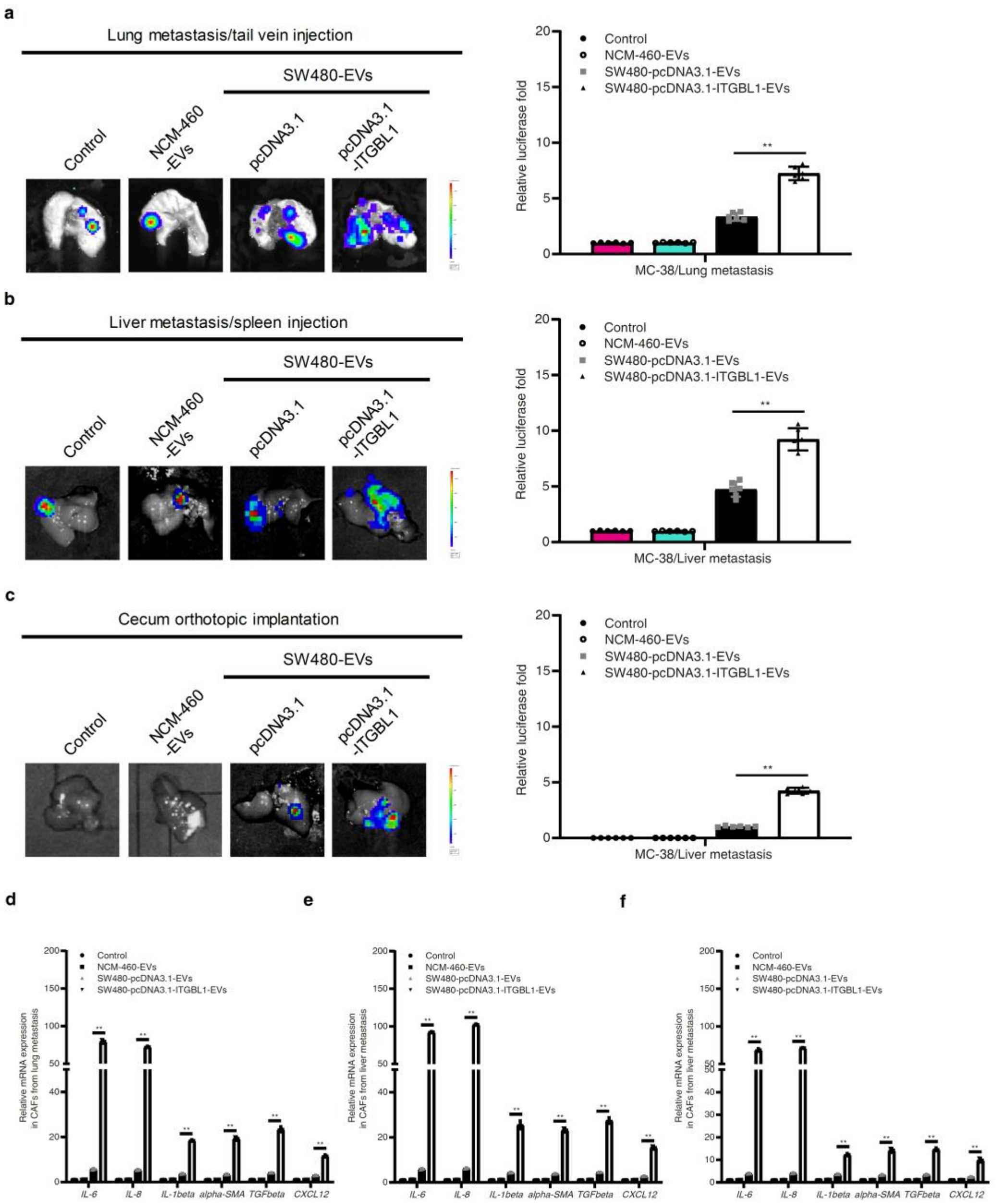

Supplementary Figure 22. EVs derived from ITGBL1-overexpressing CRC cells increased the liver and lung metastasis in C57Bl/6 mice. a Luciferase-based bioluminescence imaging on the lung of the indicated mice treated with EVs derived from normal colonic epithelial cells NCM-460, ITGBL1-overexpressing CRC SW480 cells relative to their controls, and the blank control was the MC-38 cells without EVs treatment. Luciferase labeled mouse colon cancer cell MC-38 was used to perform experimental lung metastasis model by tail vein injection. For each group, 6 mice were used for quantification. **b** Luciferase-based bioluminescence imaging on the liver of the indicated mice treated with EVs derived from NCM-460, ITGBL1-overexpressing CRC SW480 cells relative to their

controls, and the blank control was the MC-38 cells without EVs treatment. Luciferase labeled MC-38 was used to perform experimental liver metastasis model by spleen injection. For each group, 6 mice were used for quantification. **c** Luciferase-based bioluminescence imaging on the liver of the indicated mice treated with EVs derived from NCM-460, ITGBL1-overexpressing SW480 cells relative to their controls, and the blank control was the MC-38 cells without EVs treatment. Luciferase labeled MC-38 was used to perform liver metastasis model by cecum orthotopic transplantation. For each group, 6 mice were used for quantification. **d** qRT-PCR analysis of indicated gene (*IL-6*, *IL-8*, *IL-1β*, *α-SMA*, *TGF-β*, *CXCL12*) expressions in the CAFs isolated from lung metastasis in **a**. **e** qRT-PCR analysis of indicated gene expressions (*IL-6*, *IL-8*, *IL-1β*, *α-SMA*, *TGF-β*, *CXCL12*) in the CAFs isolated from liver metastasis in **b**. **f** qRT-PCR analysis of indicated gene expressions (*IL-6*, *IL-8*, *IL-1β*, *α-SMA*, *TGF-β*, *CXCL12*) in the CAFs isolated from liver metastasis in **c**. Each experiment was performed at least in triplicate and the results are shown as mean ±SD. Student's t-test was used to analyze the data. *$p < 0.05$; **$p < 0.01$.

i

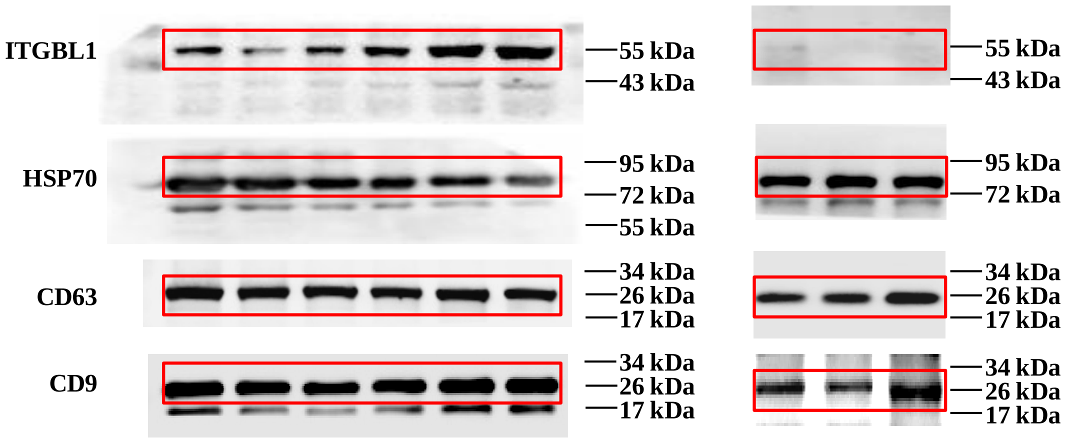

Supplementary Figure 23. Full-length uncropped western blots for Figure 1. Cropped areas are marked by red box.

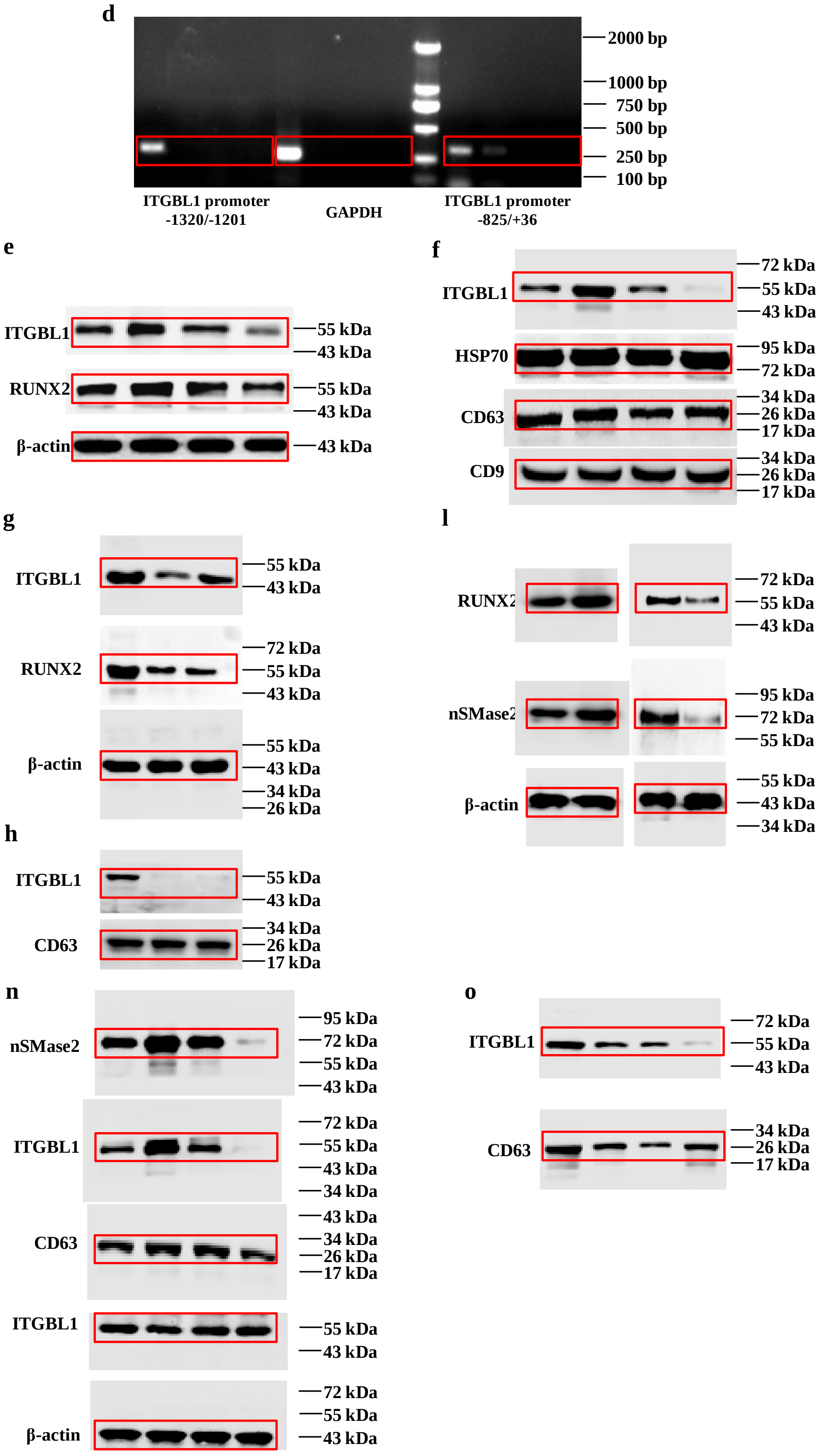

Supplementary Figure 24. Full-length uncropped gels or western blots for Figure 2. Cropped areas are marked by red box.

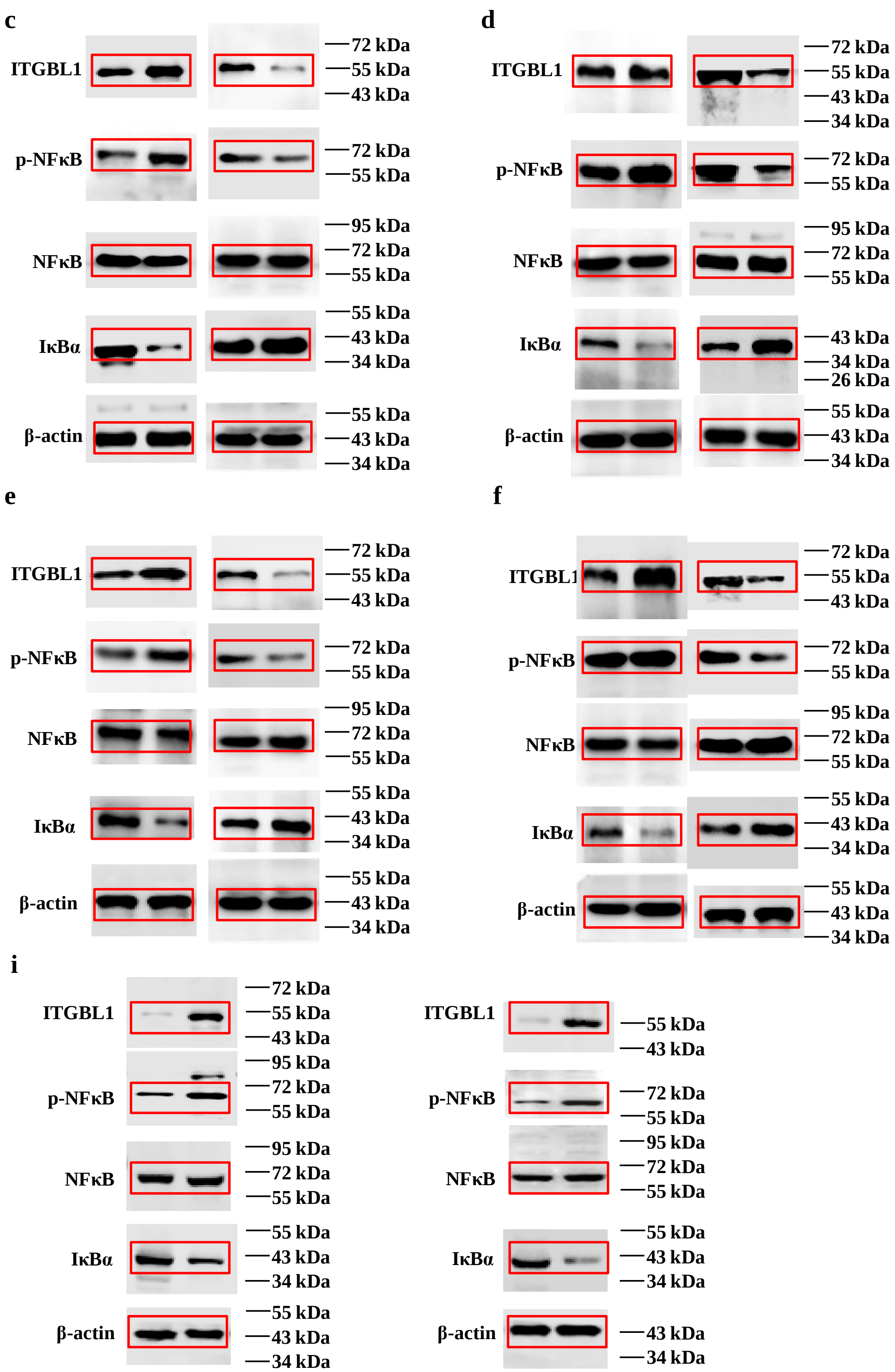

Supplementary Figure 25. Full-length uncropped western blots for Figure 5. Cropped areas are marked by red box.

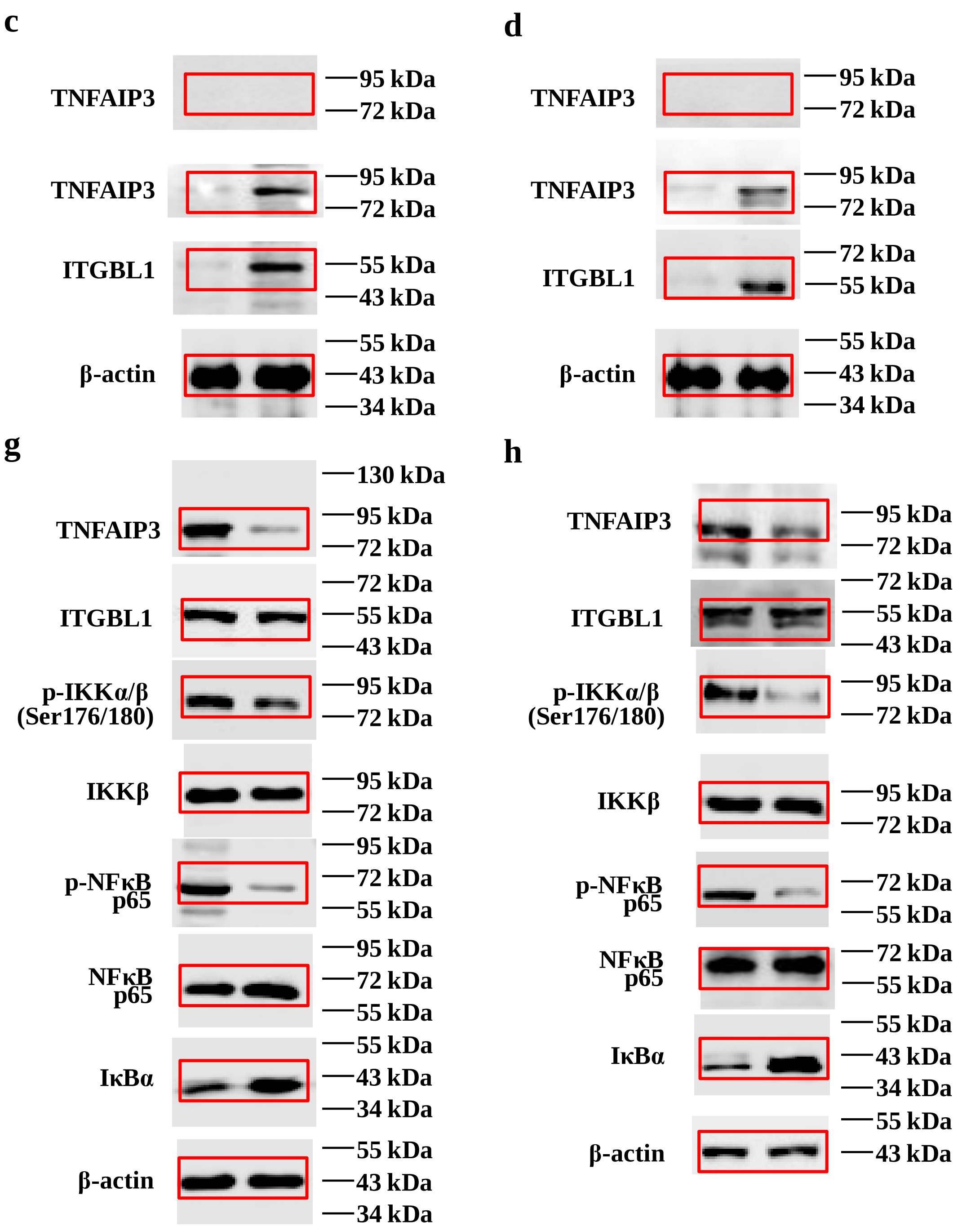

Supplementary Figure 26. Full-length uncropped western blots for Figure 6. Cropped areas are marked by red box.

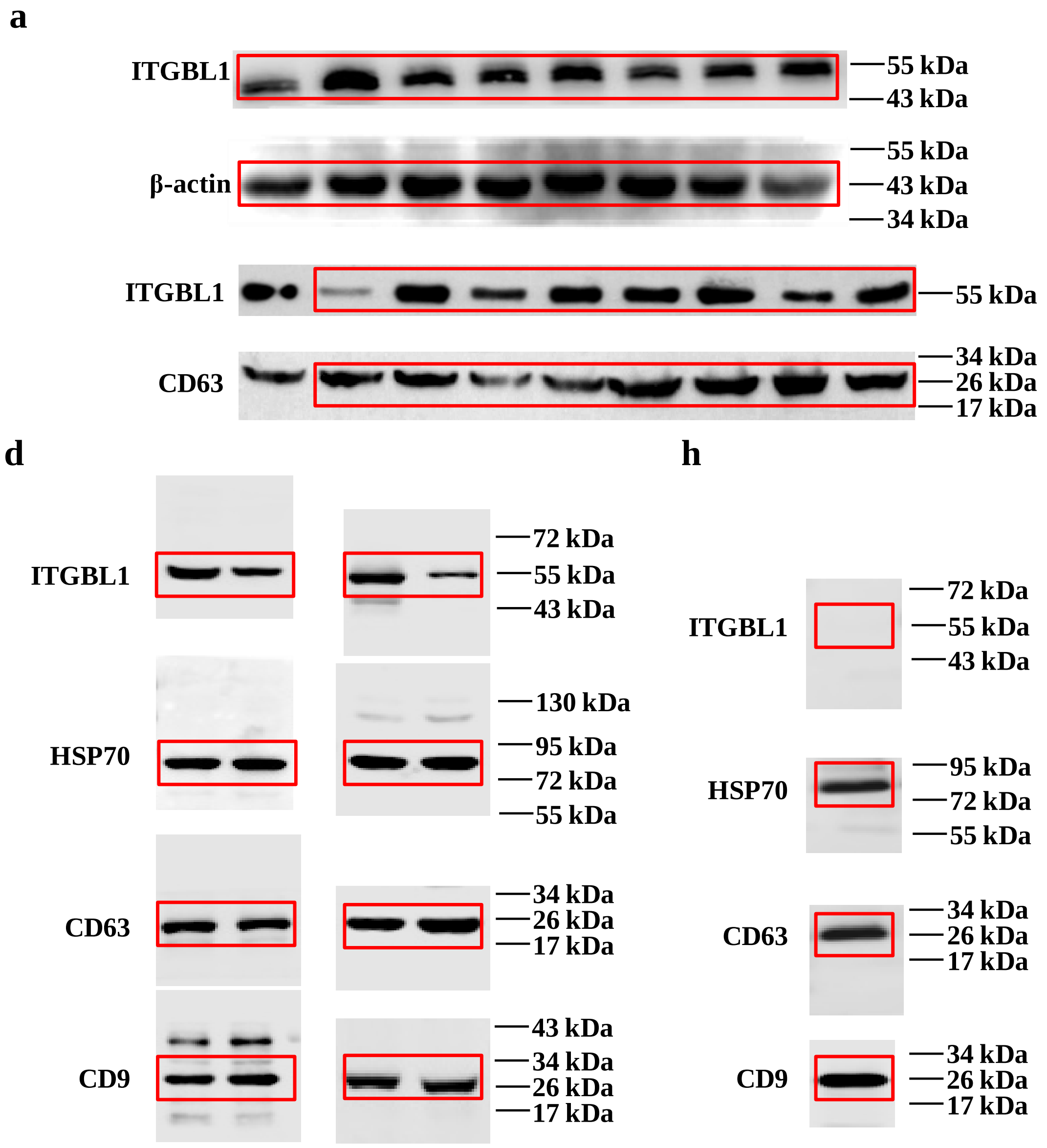

Supplementary Figure 27. Full-length uncropped western blots for Supplementary Figure 7. Cropped areas are marked by red box.

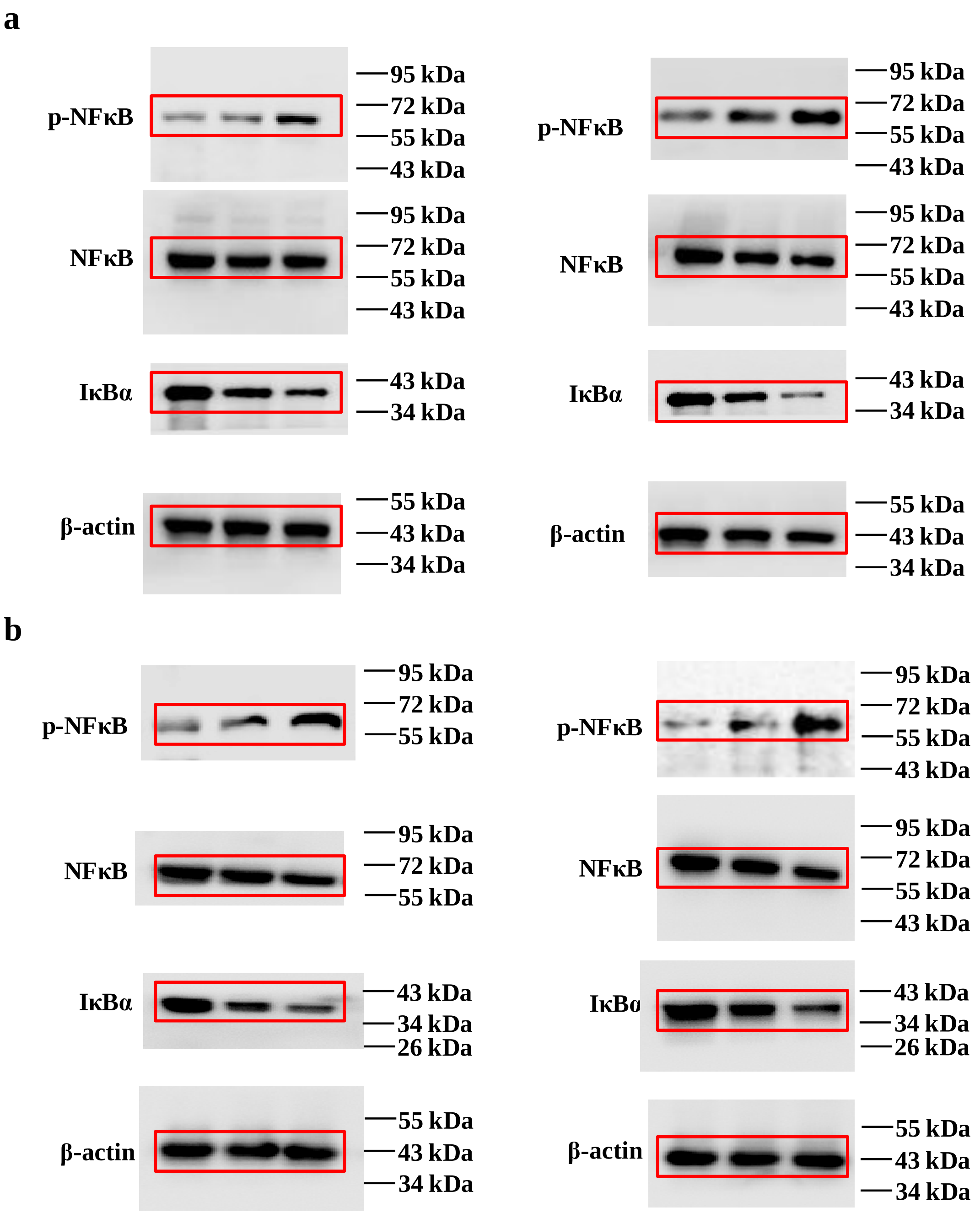

Supplementary Figure 28. Full-length uncropped western blots for Supplementary Figure 11. Cropped areas are marked by red box.

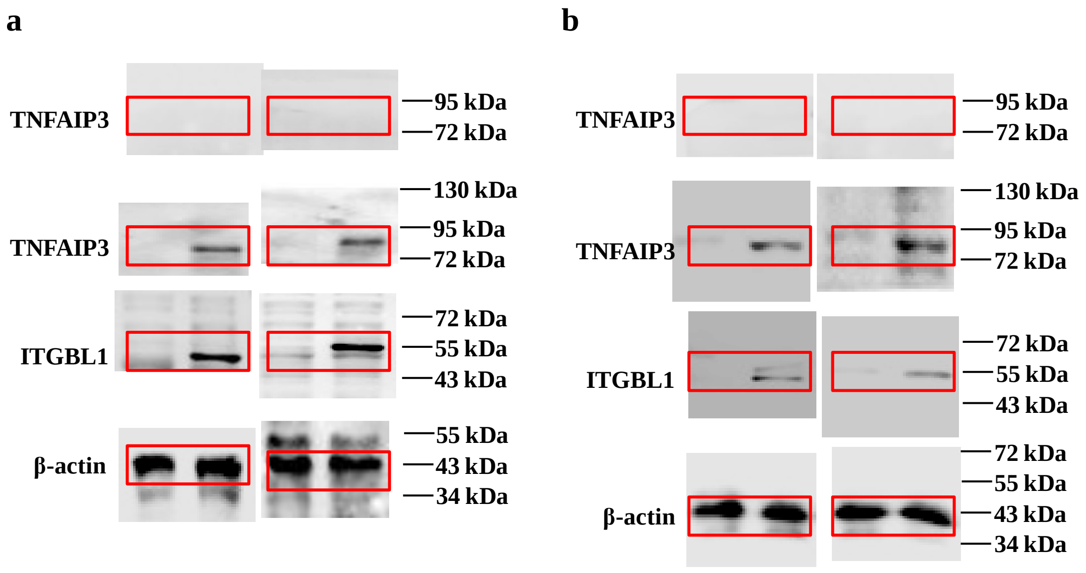

Supplementary Figure 29. Full-length uncropped western blots for Supplementary Figure 12. Cropped areas are marked by red box.

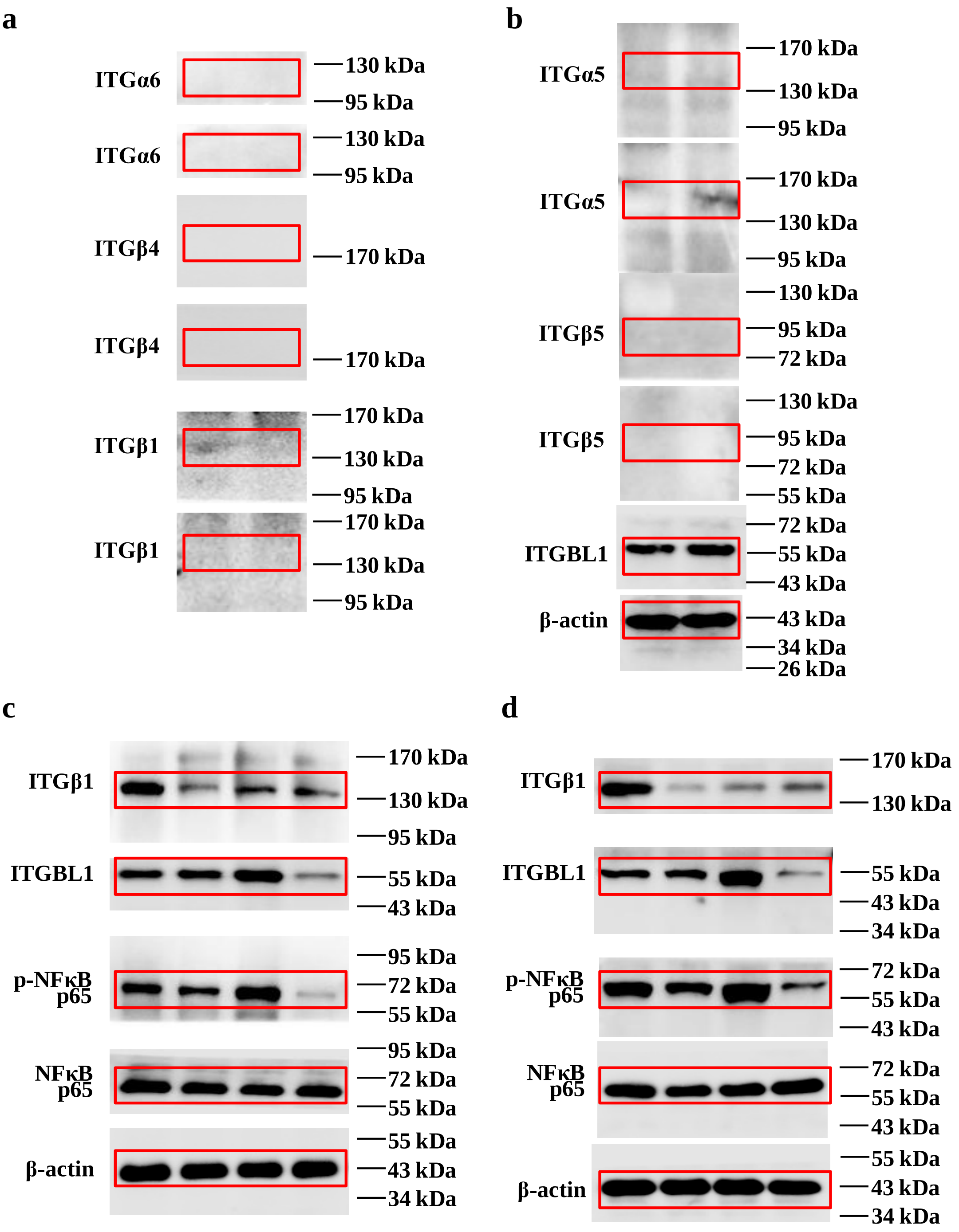

Supplementary Figure 30. Full-length uncropped western blots for Supplementary Figure 13. Cropped areas are marked by red box.

b

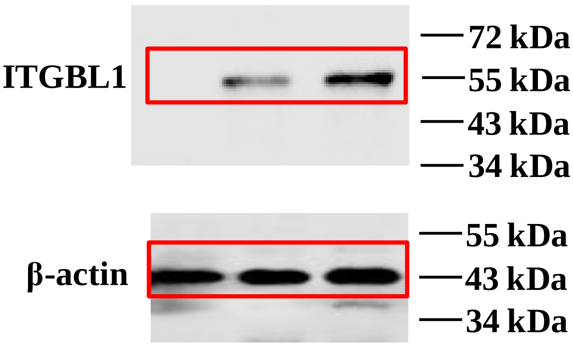

Supplementary Figure 31. Full-length uncropped western blots for Supplementary Figure 17. Cropped areas are marked by red box.

c

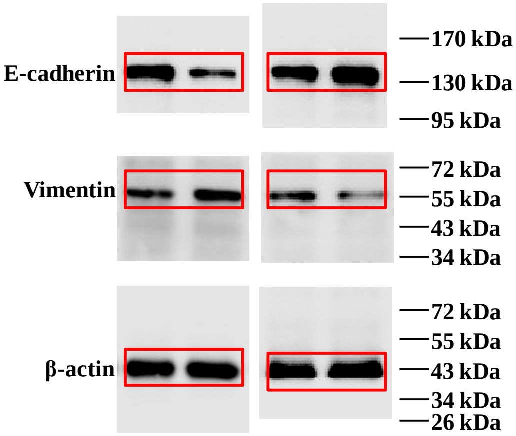

d

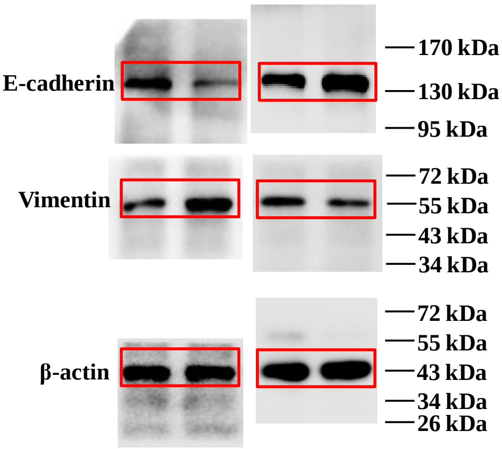

Supplementary Figure 32. Full-length uncropped western blots for Supplementary Figure 19. Cropped areas are marked by red box.

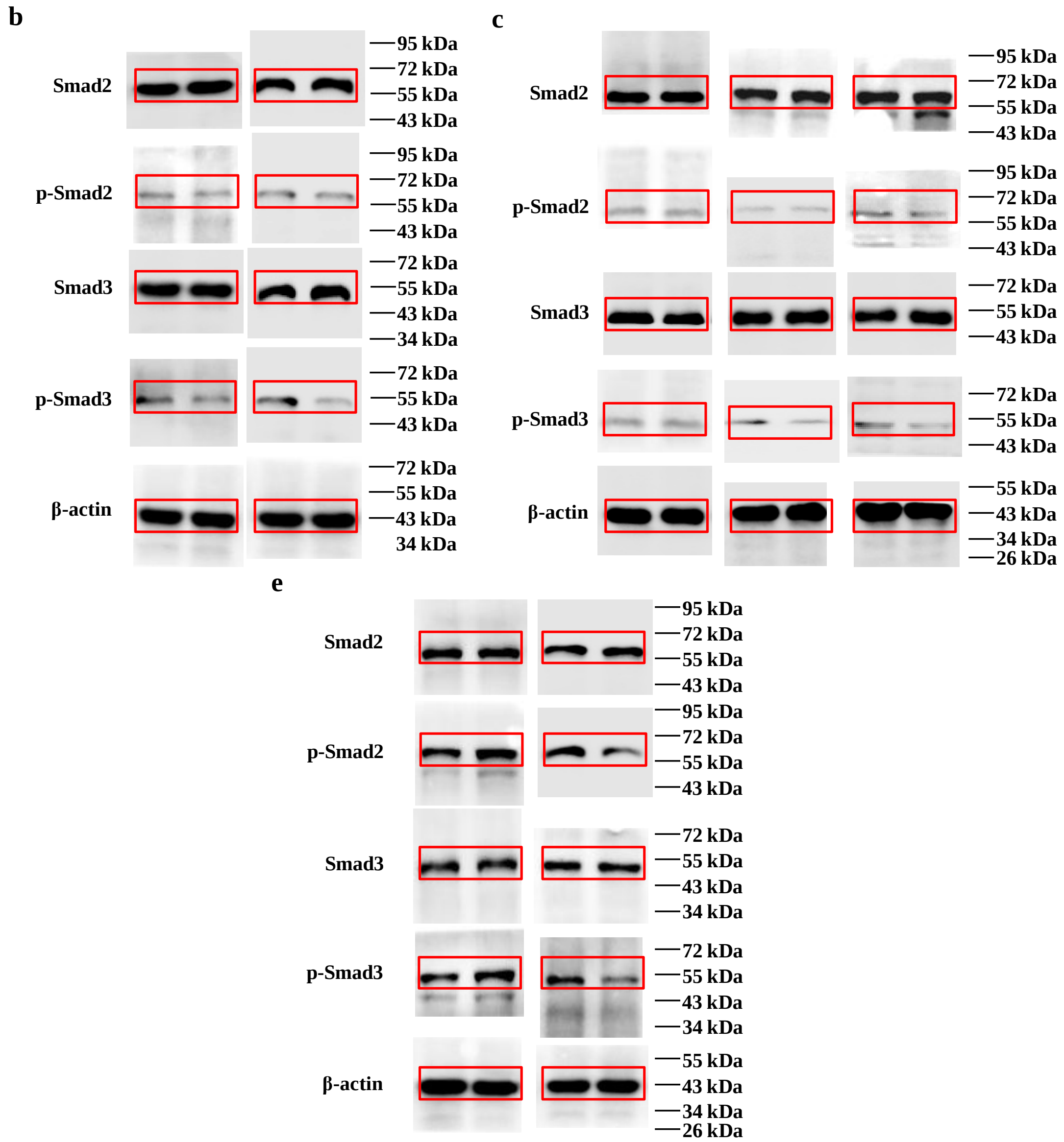

Supplementary Figure 33. Full-length uncropped western blots for Supplementary Figure 20. Cropped areas are marked by red box.

Supplementary Table 1. Association between ITGBL1 mRNA expression and clinicopathological variables of the 124 studied CRC patients

Variables	Low ITGBL1 expression (n=68)		High ITGBL1 expression (n=56)		*p* value
	n (%)		n (%)		
**Age**					
>65	15	(22.06)	8	(14.29)	0.3543
≤65	53	(77.94)	48	(85.71)	
**Gender**					
Male	45	(66.18)	33	(58.93)	0.4574
Female	23	(33.82)	23	(41.07)	
**Tumor site**					
Rectum	39	(55.35)	34	(60.71)	0.7181
Colon	29	(44.65)	22	(39.29)	
**Tumor differentiation**					
Well	20	(29.41)	19	(33.93)	0.6980
Moderate + Poor	48	(71.59)	37	(66.07)	
**TNM stage**					
Stage II	37	(54.41)	10	(17.86)	< **0.0001**
Stage III-IV	31	(45.59)	46	(82.14)	
**Lymph vascular invasion**					
Positive	47	(69.12)	39	(69.64)	1.000
Negative	21	(30.88)	17	(30.36)	
**Perineural invasion**					
Positive	58	(85.29)	40	(71.43)	0.0766
Negative	10	(14.71)	16	(28.57)	
**Liver metastasis**					
Positive	12	(17.65)	23	(41.07)	**0.0050**
Negative	56	(82.35)	33	(58.93)	
**Lung metastasis**					
Positive	2	(2.94)	9	(16.07)	**0.0223**
Negative	66	(97.06)	47	(83.93)	

Supplementary Table 2. Quantitative analysis of ITGBL1 expression detected by IHC in human CRC tissues

Tissue types	Case (n)	Staining Intensity					*p* value
		−	+/−	+	++	+++	
**Primary tumor I**	78	9	19	32	13	5	
**Primary tumor II**	46	1	4	10	17	14	<0.01
**Lung Met**	11	0	1	1	3	6	<0.01
**Liver Met**	35	1	3	7	11	13	<0.01

Note: Primary tumor I: tissues from CRC patients without paired metastatic tissues; Primary tumor II: tissues from CRC patients with paired metastatic tissues; Lung Met: lung metastatic tissues; Liver Met: liver metastatic tissues. $p<0.01$ vs Primary tumor I.

Supplementary Table 3. Multivariate analysis of clinicopathological factors in the 124 studied CRC patients

**Variables**	**Hazard ratio**	**95% CI**	***p* value**
**ITGBL1 in EVs**			
High			
Low	2.998	1.356-6.629	**0.007**
**TNM stage**			
Stage II			
Stage III-IV	3.738	1.398-8.335	**0.053**
**Distant metastasis**			
Positive			
Negative	3.668	1.751-7.683	**0.001**

Supplementary Table 4. Distribution of the included CRC patients according to clinicopathological variables

**Variables**	**Cases (%)**
**Age**	
>65	23 (18.5)
≤65	101 (81.5)
**Gender**	
Male	78 (62.9)
Female	46 (37.1)
**Tumor site**	
Rectum	73 (58.9)
Colon	51 (41.1)
**Tumor differentiation**	
Well	39 (31.5)
Moderate + Poor	85 (68.5)
**TNM stage**	
Stage II	47 (37.9)
Stage III-IV	77 (62.1)
**Lymph vascular invasion**	
Positive	86 (79.0)
Negative	38 (21.0)
**Perineural invasion**	
Positive	98 (84.7)
Negative	26 (15.3)
**Liver metastasis**	
Positive	35 (28.23)
Negative	89 (71.77)
**Lung metastasis**	
Positive	11 (8.87)
Negative	113 (91.13)

Supplementary Table 5. Primer sequences for plasmid construction

Gene	Primer sequences
ITGBL1	F: 5-CCCAAGCTTATGTGCAAGAATTC-3<br>R:5-CCGGAATTCTTAAGGATATTCTGAGCC-3
RUNX2	F: 5-CCCAAGCTTATGGCATCAAACAG-3<br>R: 5-CCGGAATTCTCAATATGGTCGCC-3
SMPD3<br>(nSMase2)	F: 5-ATGGTTTTGTACACGACC-3<br>R: 5-CTATGCCTCCTCCTCCCCCGAAC-3

Supplementary Table 6. shRNA sequences for ITGBL1, RUNX2, nSMase2, TNFAIP3 and ITGβ1 genes

**Gene**	**shRNA sequences**
ITGBL1	sense: 5-AGUCUCGAAUGAUCGUU-3<br>anti-sense: 5-AACGAUCAUUCGAGACU-3
RUNX2	sense: 5-AUCUACUGUAACUUUAAUUGCUCUG-3<br>anti-sense: 5-CAGAGCAAUUAAAGUUACAGUAGAU-3
nSMase2	sense: 5-GGAGAUUUCAACUUUGAUA-3<br>anti-sense: 5-UAUCAAAGUUGAAAUCUCCTT-3
TNFAIP3	sense: 5-AGACACACGCAACTTTAAA-3<br>anti-sense: 5-UUUAAAGUUGCGUGUGUCU-3
ITGβ1	sense: 5-GGAAAUGGUGUUUGCAAGU-3<br>anti-sense: 5-ACUUGCAAACACCAUUUCC-3
Non-targeting control shRNA	sense: 5-CCUCUAGGUAAGCAUAAUUTT-3<br>anti-sense: 5-AAUUAUGCUUACCUAGAGGTT-3

Supplementary Table 7. Primer sequences for quantitative real time PCR

Gene	Primer sequences
ITGBL1	F: 5-GGCTGGTATGGGAAGAAGTGT-3<br>R: 5-CGATCTCCTGGAGGATAGCA-3
ITGBL1<br>-promoter	F: 5-GTTGACCTCTTTGCCCTCAG-3<br>R: 5-TCAGGTGCCAGGTTTTGTGC-3
RUNX2	F: 5-CGCCTCACAAACAACCACAG-3<br>R: 5-TCACTGTGCTGAAGAGGCTG-3
IL-6	F: 5-ACTCACCTCTTCAGAACGAATTG-3<br>R: 5-CCATCTTTGGAAGGTTCAGGTTG-3
IL-8	F: 5-TTTTGCCAAGGAGTGCTAAAGA-3<br>R: 5-AACCCTCTGCACCCAGTTTTC-3
IL-1β	F: 5-ATGATGGCTTATTACAGTGGCAA-3<br>R: 5-GTCGGAGATTCGTAGCTGGA-3
α-SMA	F: 5-AGGTAACGAGTCAGAGCTTTGGC-3<br>R: 5-CTCTCTGTCCACCTTCCAGCAG-3
TGF-β	F: 5-CCCGCATCCCAGGACCTCTCT-3<br>R: 5-CGGGGGACTGGCGAGCCTTAG-3
CXCL12	F: 5-GATTCTTCGAAAGCCATGTTG-3<br>R: 5-CACTTTAGCTTCGGGTCAATG-3
SOX2	F: 5-TTGCTGCCTCTTTAAGACTAGGA-3<br>R: 5-CTGGGGCTCAAACTTCTCTC-3
OCT4	F: 5-AGTGAGAGGCAACCTGGAGA-3<br>R: 5-ACACTCGGACCACATCCTTC-3
NANOG	F: 5-CATGAGTGTGGATCCAGCTTG-3<br>R: 5-CCTGAATAAGCAGATCCATGG-3
CD133	F: 5-GCCACCGCTCTAGATACTGC-3<br>R: 5-TGTTGTGATGGGCTTGTCAT-3
E-cadherin	F: 5-TGCCCAGAAAATGAAAAAGG-3<br>R: 5-GTGTATGTGGCAATGCGTTC-3
Vimentin	F: 5-GAGAACTTTGCCGTTGAAGC-3<br>R: 5-GCTTCCTGTAGGTGGCAATC-3
Snail	F: 5-CCTCCCTGTCAGATGAGGAC-3<br>R: 5-CCAGGCTGAGGTATTCCTTG-3
Fibronectin	F: 5-CAGTGGGAGACCTCGAGAAG-3<br>R: 5-TCCCTCGGAACATCAGAAAC-3
GAPDH	F: 5-GGTGGTCTCCTCTGACTTCAACA-3

	R: 5-CCAAATTCGTTGTCATACCAGGAAATG-3
